# Decoding spatial mTOR signalling reveals distinct roles in the tumour microenvironment

**DOI:** 10.1038/s41698-026-01674-w

**Published:** 2026-09-25

**Authors:** Razan Zuhair, Mark Eastwood, Megan Bradbury, Amy Cross, Joanna Hester, Fadi Issa, Fiona Ginty, Heba Z. Sailem

**Affiliations:** 1 School of Cancer and Pharmaceutical Sciences, Stamford St., Franklin Wilkins Building, King’s College London, London, UK; 2 Tissue Image Analytics Center, University of Warwick, Coventry, UK; 3 Nuffield Department of Surgical Sciences, Level 6, John Radcliffe Hospital, University of Oxford, Oxford, UK; 4 GE HealthCare Technology and Innovation Center, Niskayuna, NY USA; 5 Department of Engineering Science, Old Road Campus Research Building, University of Oxford, Oxford, UK

**Keywords:** Biomarkers, Cancer, Computational biology and bioinformatics, Oncology

## Abstract

The tumour microenvironment comprises a variety of cell types that interact with malignant cells, influencing both cancer progression and the patient response to therapy. Given the intricate interactions among these components, assessing how signalling pathways contribute to cancer progression by acting on different components of the TME is particularly complex. Multiplexed imaging allows simultaneous assessment of signalling in multiple of these components. Here, we utilise a highly multiplexed dataset of 61 markers across 746 colorectal tumours to investigate how complex mTOR signalling in different tissue compartments influences patient prognosis. We found that mTOR pathway signalling in immune cells correlates with favourable patient survival. Advanced spatially-aware analysis of signalling activities using graph neural networks revealed that the positive association between immune mTOR signalling and patient survival was more pronounced in tumours with features of a more differentiated morphology. Integrative analysis using TCGA data and spatial transcriptomics confirmed a role for immune mTOR signalling in immune activation, accompanied by reduced TGF-β signalling. Our work highlights the importance of adopting a personalised approach to targeting mTOR in cancer patients and establishes a framework for studying cell type-specific signalling in the TME.

## Introduction

Recent advances in multiplex tissue imaging provide an unprecedented opportunity for studying complex cell signalling, permitting the assessment of multiple components in the tumour and tumour microenvironment (TME) concurrently at a single-cell level^1–4^. For example, the identity of different cell types can be visualised in the context of their spatial organisation, signalling state and morphologies. This capability allows profiling signalling activities in various interacting cell types, including cancer, immune and stromal cells, which provides a deeper understanding of how heterocellular cell signalling drives cancer progression and impacts patient outcome. Such understanding is crucial for designing effective targeted treatment strategies that account for the interaction between different TME components.

The mTOR pathway integrates nutritional and environmental cues to modulate cell proliferation, metastasis, and metabolism. It has a multifaceted influence on the TME by inducing angiogenesis and immune chemotaxis^5–7^. The activation of mTOR leads to the activation of ribosomal protein S6 and the inhibition of eukaryotic translation initiation factor 4EBP1 through their phosphorylation. S6 phosphorylation has been associated with worse patient prognosis^8,9^. There have been conflicting results on the impact of 4EBP1 phosphorylation in tumour cells on survival, as it has been associated with both worse and better prognosis in different cancers, with increasing evidence of anti-tumour role in gastric cancers^9–12^. Such variability could be due to the different roles that mTOR plays within the TME, influencing cancer cells and immune cells in diverse ways, thereby affecting overall patient outcomes.

In addition to the role of mTOR in cancer cells, the mTOR pathway plays a vital role in immune response and immune cell proliferation and differentiation, including Tregs, cytotoxic T-cells, macrophages, neutrophils and B-cells^13–18^. However, the effects of mTOR on cancer progression based on simultaneous analysis of its signalling in cancer cells and the surrounding microenvironment are underexplored. Moreover, questions about the effects of mTOR signalling in immune cells on cancer cell signalling and fate remain largely unanswered. In this study, we hypothesised that 4EBP1 phosphorylation in immune cells and its spatial distribution in tumours may affect local cancer cell biology and patient prognosis.

Representing tumours as a network of interacting cells is an effective strategy for studying spatial protein signalling as it can consider spatial relationships between cell types, morphology and tumour-microenvironment interactions. Graph neural network (GNN) is particularly suited for learning from graph-structured data as it takes a graph or a network as an input and predicts a defined output, such as signalling states or patient outcomes^19^. GNNs inherently capture the interacting nature of cells through a message-passing procedure. This allows capturing several levels of influence, as at each GNN layer, cells update their state to reflect their own state but also their neighbours’ state. After each iteration, the states of each cell’s neighbours also incorporate the states of their neighbours, which enable capturing short- and long-range cellular communications. Another notable advantage of GNNs over traditional deep learning approaches is explainability, as they allow for the identification of the contributions of cellular subpopulations to specific signalling states.

In this study, we utilised multiplexed imaging data of 746 colorectal tumours to profile mTOR signalling, spatial distribution and cellular interactions. In addition to various proteins in the mTOR pathway (e.g., 4EBP1, S6), we also included proteins related to key cancer pathways, including p53, EGFR, Myc, and HER2, to determine interconnected signalling cascades. We first performed compartmentalised image analysis of protein expression in epithelial, immune and stromal cells to investigate how mTOR signalling in these different cell types affects patient outcomes. Using an explainable graph neural network to effectively interrogate the spatial relationships in single-cell signalling data, we identified an association between 4EBP1 phosphorylation, immune activation and lower tumour stemness. These observations were validated using spatial transcriptomics. Our results underscore the importance of analysing different cell types and spatial protein expression to understand complex signalling in the TME.

## Results

### Compartmentalised image analysis reveals differential mTOR association with patient prognosis

**Fig. 1:**
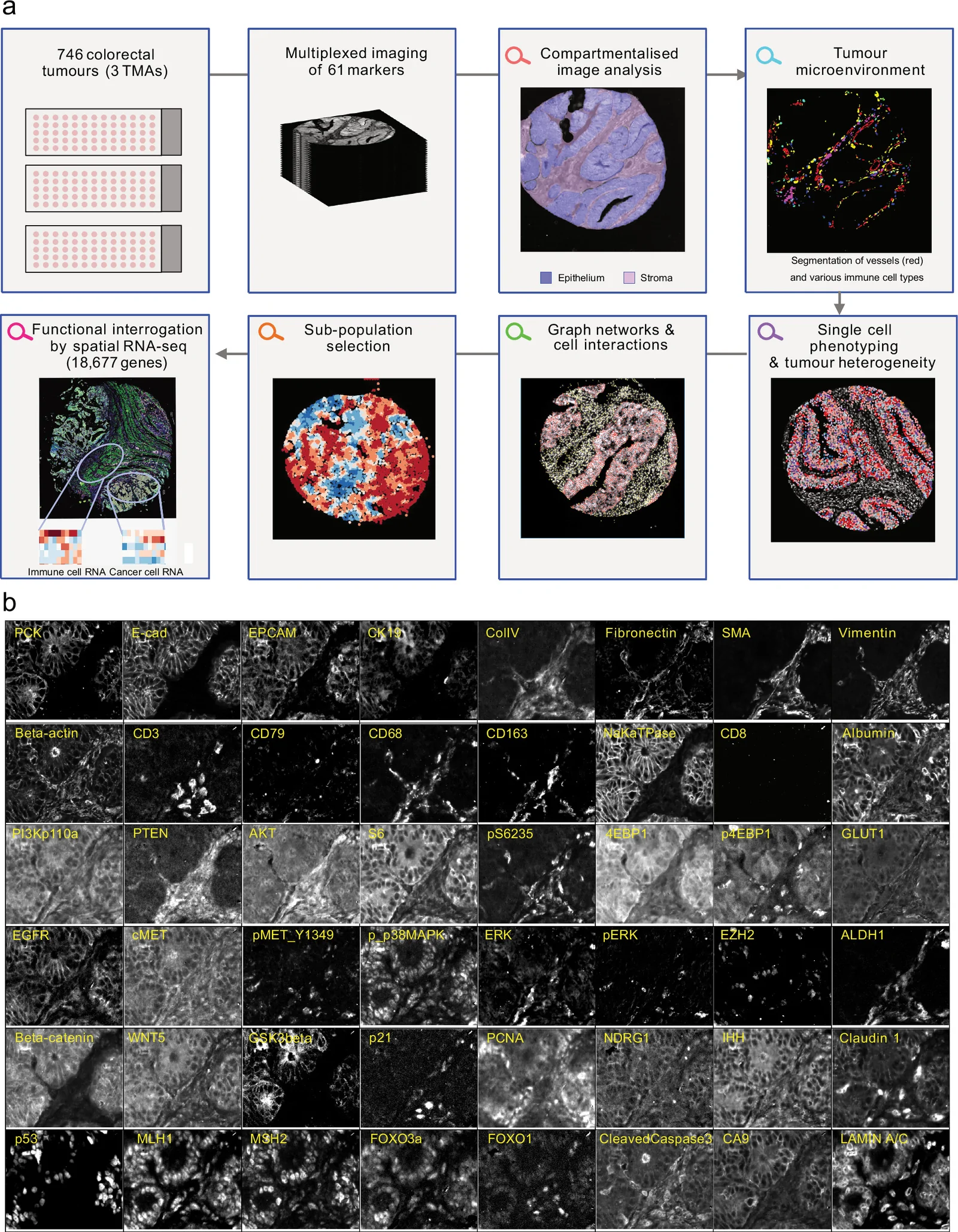
Multiplexed imaging allows multi-level investigation of complex cell signalling. **a** A schematic overview of the study, where many of the indicated analyses can be performed concurrently. Tumour microarray (TMA) layout is for representation only and does not indicate the actual TMA layout. Immune mTOR status in tumours is evaluated based mainly on p4EBP1 and pS6, where a tumour is considered positive if a certain percentage of cells express the investigated marker. Similar analyses were performed for other compartments. **b** Example images from the multiplexed dataset utilised in this study showing different markers from a selected tumour. Markers with no detectable expression in this tumour were omitted.

To investigate mTOR signalling and TME phenotypes in colorectal cancer, we utilised previously published highly multiplexed data where cyclic immunofluorescence was used to image the levels of 61 protein markers in 746 colorectal tumours with different stages and grades^1^ (Fig. 1a, b and ‘Methods’). These proteins encompassed various structural and TME markers, including epithelial proteins (pan-cytokeratin (PCK), E-cadherin, EPCAM and beta-actin), extracellular matrix (fibronectin and collagen), stroma (smooth muscle actin (SMA) and vimentin) and immune cell types (CD3, CD8, CD20, CD68, and CD79). Additionally, samples were stained for several markers in the mTOR pathway, including 4EBP1, phosphorylated 4EBP1 (p4EBP1), S6, phosphorylated S6 (pS6) and the upstream signalling components, including AKT, PTEN, p38, EGFR, MET and ERK. (Fig. 1b and Supplementary Table 1).

**Fig. 2:**
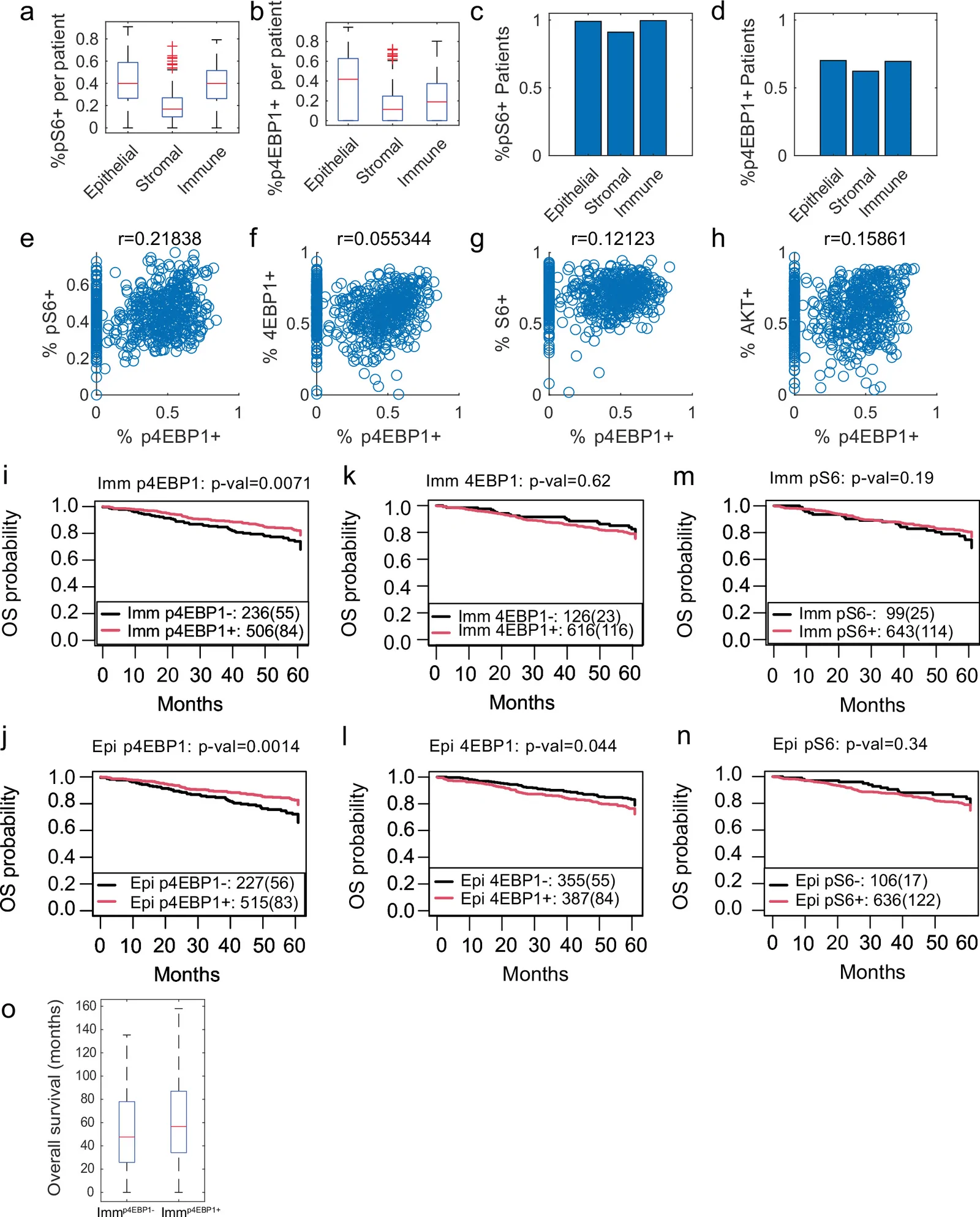
Analysis of mTOR activity in different tissue compartments and its association with survival. **a**, **b** The distribution of the percentage of positive pS6 (**a**) and p4EBP1 (**b**) cells across different tumours. **c**, **d** The proportion of tumours that are positive for: pS6 (**c**) and p4EBP1 (**d**) where a tumour is considered positive for these markers if at least 5% cells were positive in that tumour. **e**–**h** The relationship between the percentage of p4EBP1+ cells and percentages of: pS6+ cells (**e**), 4EBP1+ cells (**f**), S6+ cells (**g**), or AKT+ cells (**h**). The Pearson correlation coefficient is indicated at the top. **i** Association between overall survival (OS) and immune signalling activity based on Kaplan Meier analysis of p4EBP1+ tumours. **j** Same as (**i**) but for epithelial p4EBP1+ tumours. **k** Association between OS and immune signalling activity based on Kaplan Meier analysis of 4EBP1+ tumours. **l** Same as (**k**) but for epithelial 4EBP1+ tumours. **m** Association between OS and immune signalling activity based on Kaplan Meier analysis of pS6+ tumours. **n** Same as (**m**) but for epithelial pS6+ tumours. Black lines indicate marker-negative tumours while red lines indicate marker-positive tumours in the immune (**i**, **k**, **m**) or in the epithelial compartment (**j**, **l**, **n**). **o** Patients with Imm^p4EBP1+^ tumours survive 9 months longer than patients with Imm^p4EBP1-^ tumours. Box plots show the median (red line), interquartile range (box), whiskers extending to data points within 1.5 times the interquartile range, and outliers plotted individually (red plus markers). Imm: immune, Epi: epithelial.

We utilised the expression of pS6 and p4EBP1, respectively, as indicators of mTOR pathway activation. To systematically assess mTOR signalling across different cell types in the tumour, we classified epithelial, stromal and immune cell types based on expression of the epithelial marker (PCK), stromal cell marker (SMA) and the immune markers (CD3, CD8, CD20, CD68, CD79) (Fig. 1a and ‘Methods’). Then we identified cells that were positive for pS6, p4EBP1, or both (‘Methods’). On average, pS6 was expressed in 40% of epithelial cells, 20% of stromal cells and 40% of immune cells across 746 tumours (Fig. 2a). Similarly, p4EBP1 was expressed in 40% of epithelial cells, 15% of stromal cells and 20% of immune cells per tumour, on average (Fig. 2b). Activation of mTOR was broadly comparable across different types of immune cells (Supplementary Fig. 1a). Notably, mTOR activity varied between tumours. While most tumours had pS6 positive (pS6+ ) cells in different tissue compartments, only 62%-70% of tumours had p4EBP1 positive (p4EBP1+ ) cells (Fig. 2c, d). The lack of 4EBP1 phosphorylation in some tumours cannot be explained based on the level of pS6, S6, 4EBP1, or AKT, as low correlation was observed (Fig. 2e–h). These results indicate that while mTOR is most active in epithelial cancer cells, it is also frequently active in immune and stromal cells and that p4EBP1 levels vary between tumours.

To determine the impact of mTOR activity in different cell types on patient outcomes, we performed a Kaplan-Meier survival analysis. We found that expression of p4EBP1 in immune cells (Immp^4EBP1+^) was associated with better overall survival, with a hazard ratio of 0.63 (37% reduction in hazard) compared to the negative group (Imm^p4EBP1-^) (*p*-value = 0.0071 and Fig. 2i). Specifically, patients with Imm^p4EBP1+^ tumours have 9 months longer overall survival than patients with Imm^p4EBP1-^ tumours (median survival 56.6 versus 47.6 respectively and Fig. 2o). Similarly, p4EBP1 expression in epithelial cancer cells (Epi^p4EBP1+^), but not in fibroblasts was also associated with better overall survival (Epi^p4EBP1+^: *p*-value = 0.0014, hazard ratio=0.57 Fig. 2i, j and Supplementary Fig. 1b). These associations were consistent across several cut-offs defined based on the percentage of p4EBP1+ positive cells (FDR < 0.05 and ‘Methods’ and Supplementary Fig. 1c–f). Consistent with previous studies, 4EBP1 expression is associated with poorer outcomes only when epithelial cancer cell expression was considered (*p*-value = 0.044 Fig. 2k, l). We did not observe an association between pS6 expression and survival (Fig. 2m, n). As the phosphorylated form of 4EBP1 inhibits its action on EIF4, we investigated whether p4EBP1 association with survival is altered when combined with 4EBP1 expression levels. We observed that expression of 4EBP1 in the immune compartment does not affect the association between Imm^p4EBP1+^ tumours and survival (Supplementary Fig. 1e). On the other hand, the association between epithelial p4EBP1 and survival diminishes when 4EBP1 is also expressed in epithelial cancer cells even at low levels (Supplementary Fig. 1f). These results suggest that inhibition of 4EBP1 through its phosphorylation could provide prognostic advantage and its association with survival in epithelial cells is dependent on the levels of unphosphorylated 4EBP1.

We further examined the association between mTOR and various clinical variables. The proportion of Imm^p4EBP1+^ tumours did not associate with age or grade (Supplementary Fig. 1g). However, we observed a significant association between Imm^p4EBP1+^ tumours and stage (chi-square test *p-*value = 5.8678e-09). Specifically, the proportion of Imm^p4EBP1+^ tumours was highest in Stage II but lowest in Stage III tumours (Supplementary Fig. 1g). Cox multivariate regression analysis showed that the association between Imm^p4EBP1+^ and survival was independent of grade, gender, or age but not of tumour stage (Supplementary Table 2). Nonetheless, the Wald test indicated that even though Imm^p4EBP1+^ association with survival was not independent of tumour stage, these two variables collectively have a highly significant effect on survival with high concordance (Wald test *p-*value = 1.2E-12, concordance = 0.69).

### Characterisation of signalling and TME in Imm^p4EBP1+^ tumours

**Fig. 3:**
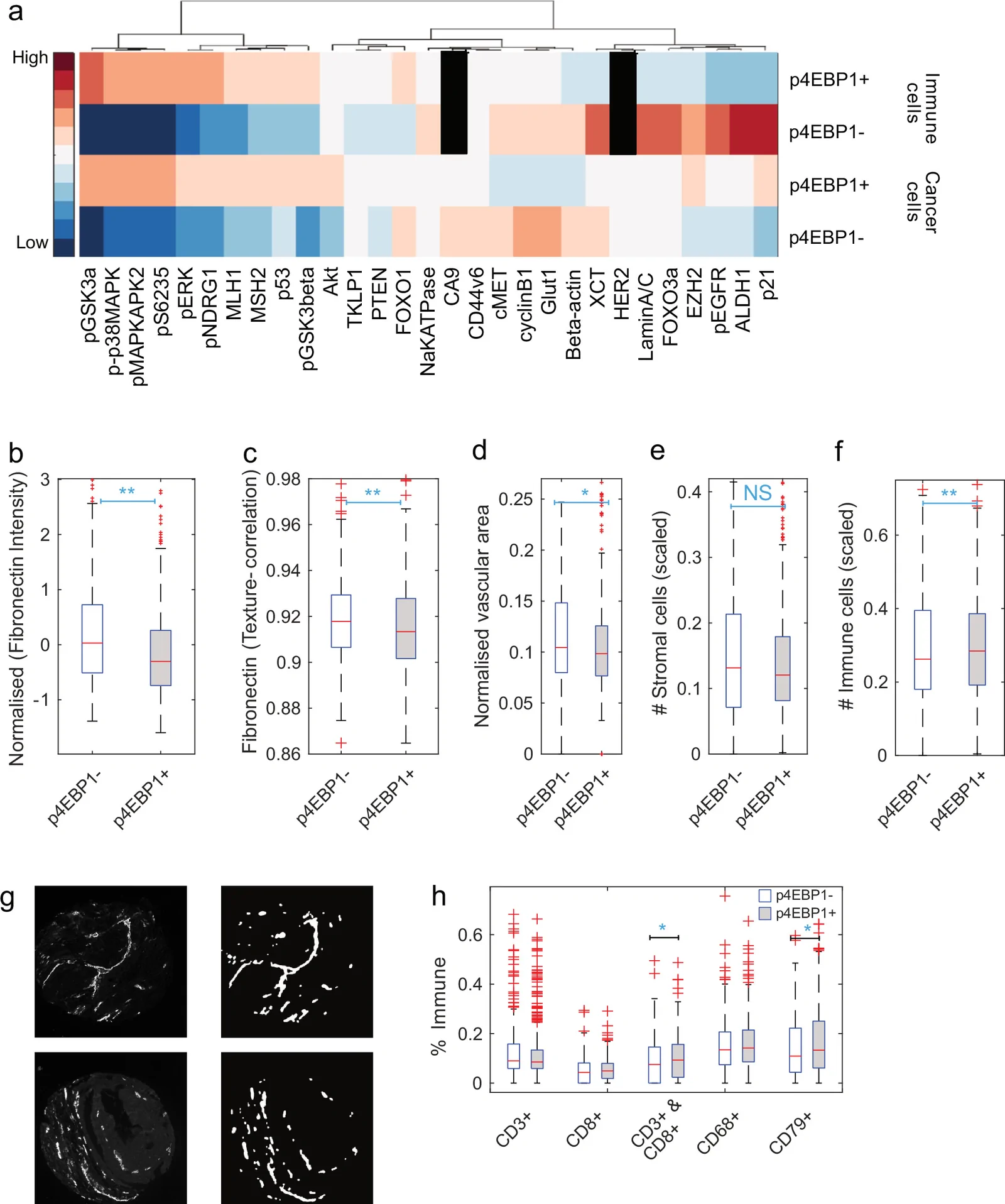
Cell signalling and TME properties in Imm^p4EBP1+^ tumours. **a** Top differentially expressed proteins between Imm^p4EBP1-^ and Imm^p4EBP1+^ tumours in both the immune and epithelial compartments. Black rectangles indicate markers that are not known to be expressed in immune cells (‘Methods’). The colour scale ranges from blue (low marker expression values) to red (high marker expression values). **b** Fibronectin levels in Imm^p4EBP1-^ versus Imm^p4EBP1+^ tumours based on normalised intensity (Kolmogorov–Smirnov (KS) *p-*value = 1.4e-5). **c** Haralick correlation between pixels in fibronectin images in Imm^p4EBP1-^ versus Imm^p4EBP1+^ tumours (KS *p-*value = 0.0013). **d** Difference in vascular area in Imm^p4EBP1-^ versus Imm^p4EBP1+^ tumours (KS *p-*value = 0.017). **e** Number of stromal cells in Imm^p4EBP1-^ versus Imm^p4EBP1+^ tumours. NS indicates no significant difference. **f** Number of immune cells in Imm^p4EBP1-^ versus Imm^p4EBP1+^ tumours (KS *p-*value = 0.0083). **g** Example images showing vessel segmentation (right) for measuring vessel area based on the CD31 marker (left). **h** Percentage of different immune cell types in Imm^p4EBP1-^ versus Imm^p4EBP1+^ tumours. A star indicates a KS *p-*value < 0.05, while two stars indicate *p*-value < 0.01. Box plots show the median (red line), interquartile range (box), whiskers extending to data points within 1.5 times the interquartile range, and outliers plotted individually (red plus markers), where grey boxes indicate Imm^p4EBP1+^ tumours.

To identify the signalling activities associated with Imm^p4EBP1+^ tumours, we investigated differentially expressed proteins in tumours with p4EBP1+ /- immune cells. The top 30 significantly expressed proteins were selected (‘Methods’). Protein levels of LaminA/C, FOXO3a, EZH2, pEGFR, ALDH1, and p21 in immune cells were significantly lower in Imm^p4EBP1+^ tumours. However, the expression of these proteins in cancer cells was similar (Fig. 3a). Imm^p4EBP1+^ tumours had lower levels of cMET, CyclinB1 and Glut1, beta-actin, and xCT in both immune and epithelial cells. Furthermore, Imm^p4EBP1+^ tumours exhibited higher levels of ERK and downstream signalling of MAPK such as pGSK3α, p38MAPK, pMAPAPK2, pS6235, pERK, NDRG1, MLH1, MSH2, p53, and GSKβ in both epithelial and immune cells. Together, these findings indicate that Imm^p4EBP1+^ tumours are associated with both immune-compartment-specific changes and shared epithelial-immune signalling, including higher levels of ERK/MAPK- and DNA-damage-response-associated proteins.

Next, we compared the TME between Imm^p4EBP1+^ and Imm^p4EBP1-^ tumours. We found that fibronectin and vascularisation were significantly reduced in Imm^p4EBP1+^ tumours (Fig. 3b–g and ‘Methods’). These changes were not due to differences in the number of stromal cells (Fig. 3e). We also observed a significant increase in the total number of immune cells, specifically CD79+, and CD3+ CD8+ cells (*p-*value < 0.05 and Fig. 3f, h). These results suggest that p4EBP1 expression in immune cells is associated with lower fibronectin expression.

### Graph neural network for spatially aware analysis of mTOR signalling in immune cells

**Fig. 4:**
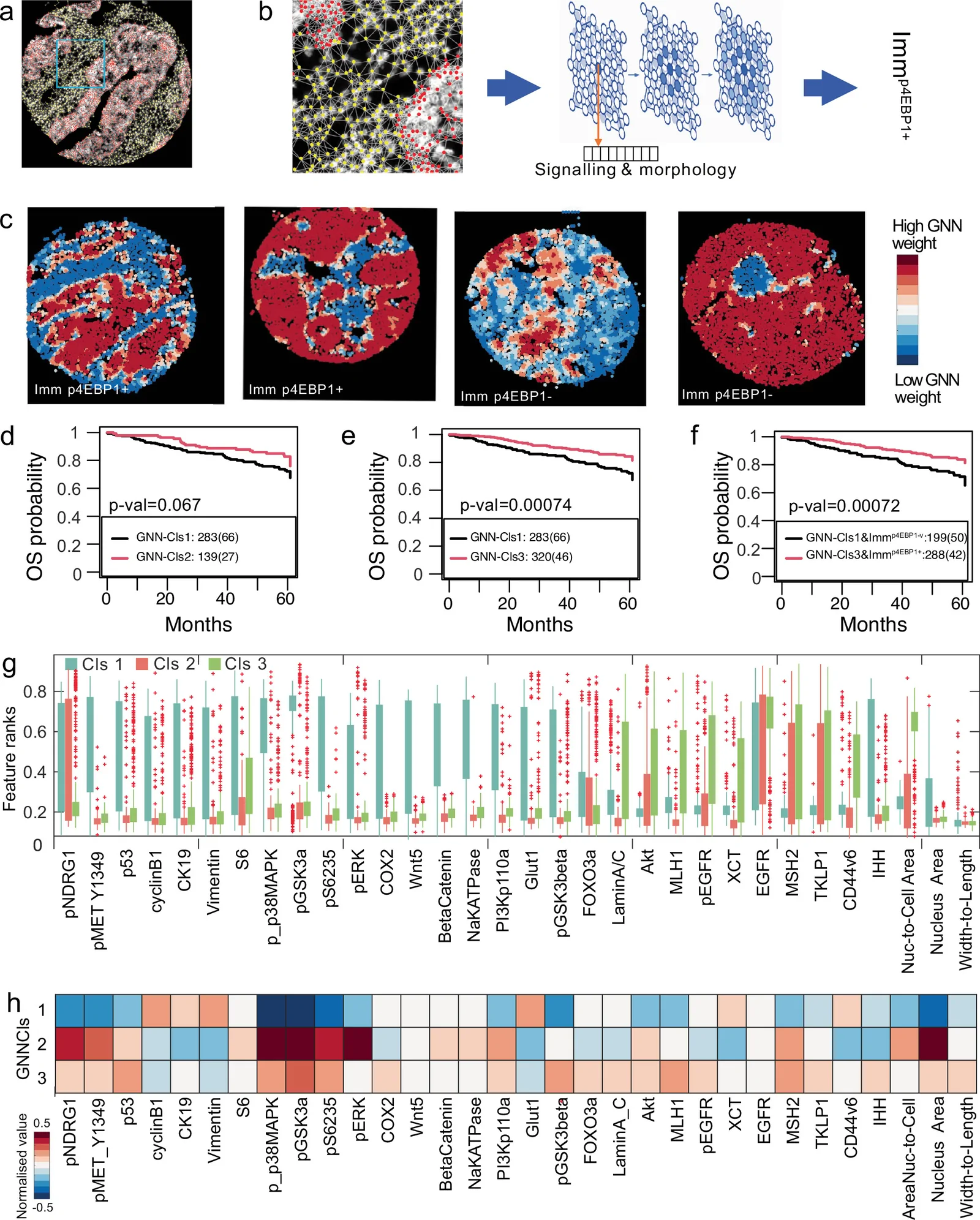
Prediction of Imm^p4EBP1+^ tumours and identification of relevant cellular subpopulation using graph neural networks. **a** Cells are projected as a network based on their spatial distance, where red nodes indicate epithelial cells and yellow indicate stromal and immune cells. **b** Representation of graph neural network (GNN) classifier through message passing between cells to predict Imm^p4EBP1-^ versus Imm^p4EBP1+^ tumours. **c** Visualisation of node/cell scores, which were used to identify high-scoring cells. Shown are the scores contributing to the predicted class (Imm^p4EBP1+^ versus Imm^p4EBP1-^) based on weights learned by GNN for 2 Imm^p4EBP1+^ tumours and 2 Imm^p4EBP1-^ tumours. Red indicates a positive contribution, and blue indicates a negative contribution to the predicted class. **d**, **e** Association between overall survival (OS) and GNN-Cls 1 (black line) versus GNN-Cls 2 (red line) (**d**) or versus GNN-Cls 3 (red line) (**e**) using Kaplan–Meier survival analysis. **f** Same as (**e**), but comparingGNN-Cls 1 Imm^p4EBP1-^ tumours with GNN-Cls 3 Imm^p4EBP1+^ tumours. **g** Feature ranks for the top selected features identified by GNNExplainer across GNN-Cls 1 (cyan boxes), GNN-Cls 2 (red boxes), and GNN-Cls 3 (green boxes). Box plots show interquartile range (box), whiskers extending to data points within 1.5 times the interquartile range, and outliers plotted individually (red plus markers). **h** Actual expression values of features shown in (**g**) in the epithelial compartment. The colour scale ranges from blue (low marker expression) to red (high marker expression).

To account for spatial cell signalling and cell-type interactions, we utilised a graph neural network (GNN) to construct a cell network representing the spatial relationships between cells in the tumour^20^. Specifically, we defined cells as nodes within a network, with edges representing interactions between neighbouring cells within a 50-pixel radius (Fig. 4a). Each cell node in the graph incorporated features representing its signalling, context, and morphological measurements (‘Methods’). This network was then fed into a GNN to predict Imm^p4EBP1+^ versus Imm^p4EBP1-^ tumours (Fig. 4b and ‘Methods’). We achieved an Area Under the ROC Curve (AUC) of 82%, an Average Precision (AP) of 91%, and an accuracy of 75% (‘Methods’ and Supplementary Fig. 2a). Despite that p4EBP1 levels and status at single-cell levels were not included in the features that were used for training GNN, we found that 75.05% of Imm^p4EBP1+^ cells were assigned a high weight by GNN (Fig. 4c). These cells represent only 8.95% of high-scoring cells (‘Methods’). Other high-scoring cells constituted 17.18% immune, 7.4% stromal and 66.47% epithelial cells. This provided confidence that the network detected relevant signatures in Imm^p4EBP1+^ cells. It also suggests that phosphorylation of 4EBP1 in immune cells is associated with complex interactions between the tumour and immune cells.

We utilised GNNExplainer^20^ to weigh the contribution of each feature to the prediction of Imm^p4EBP1+^ tumour status (‘Methods’). Clustering of tumours based on top-ranked features by GNN identified that tumours with and without Imm^p4EBP1+^ cells can be distinguished based on different sets of features (Supplementary Fig. 2b). GNN-based clustering also revealed that tumours with Imm^p4EBP1+^ cells can be divided into two subgroups (GNN-Cls 2 and GNN-Cls 3) (Supplementary Fig. 2c). These two clusters were generally similar in terms of the number of Imm^p4EBP1+^ cells, accuracy, GNN confidence, and the number of high-scoring cells (Supplementary Fig. 2d-g). GNN-Cls 2 was not significantly associated with overall survival (p = 0.067, Fig. 4d). On the other hand, GNN-Cls 3 was highly associated with favourable overall survival (p-value = 0.0007, hazard ratio=0.53 and Fig. 4e). This is more significant when compared to *p*-value of 0.007 when both GNN-Cls 2 and 3 were combined (Fig. 2i). As GNN-Cls 3 included a subset of patients with Imm^p4EBP1-^ tumours (10% of patients), we compared GNN-Cls 3 and Imm^p4EBP1+^ tumours versus GNN-Cls 1 and Imm^p4EBP1-^ tumours to determine whether the improved stratification was specifically attributable to the p4EBP1 signature (Supplementary Fig. 2c). This refined grouping resulted in a comparable *p*-value of 0.0007 (Fig. 4f). Consistent with this finding, multivariable Cox regression confirmed the independent prognostic value of the GNN-Cls grouping (*p*-value = 0.02 and Supplementary Table 2). Importantly, GNN-Cls 2 and 3 were not associated with tumour stage or grade (Supplementary Fig. 2h, i). Thus, GNN allows further stratification of tumours with Imm^p4EBP1+^ cells based on a holistic analysis of spatial protein activity.

To further understand the association between p4EBP1 activity in immune cells and patient outcome, we compared the ranking and values of the top predictive features across GNN clusters based on GNNExplainer (Fig. 4g-h). In GNN-Cls 1, phospho-p38, pMET, PI3Kp110a, NaKaTPase and the adhesion protein BetaCatenin received the highest ranks by GNNExplainer, with expression values generally lowest in GNN-Cls 1, highest in GNN-Cls 2, and intermediate in GNN-Cls 3 (Supplementary Table 3). Only a few features have a high ranking for GNN-Cls 2, and these overlapped with GNN-Cls 3. In GNN-Cls 3, predictive markers include xCT, MSH2, MLH1, pEGFR, EGFR, and AKT (Fig. 4g). Interestingly, markers of stemness such as CD44 and Lamin A/C, as well as morphological features, such as the ratio of nuclear-to-cell area exhibited the highest ranks for only GNN-Cls 3 (Fig. 4g). Investigating the actual expression of these markers revealed that GNN-Cls 2 showed stronger pS6, pERK/MAPK-related, pNDRG1, pGSK3α signalling (Fig. 4h). In contrast, GNN-Cls 3 exhibited elevated pGSK3β, FOXO3a, LaminA/C, p53 and MLH1 compared to GNN-Cls 2. Moreover, GNN-Cls 3 exhibited a more differentiated morphology than GNN-Cls 2, characterised by a lower nuclear-to-cell area ratio and higher width-to-length ratio, indicating lower elongation (*p*-value < 0.05). These results suggest that GNN-Cls 2 and GNN-Cls 3 show distinct Imm^p4EBP1+^-associated signalling states and morphologies.

### Determination of the functional relevance of Imm^p4EBP1+^ signalling using spatial transcriptomics

**Fig. 5:**
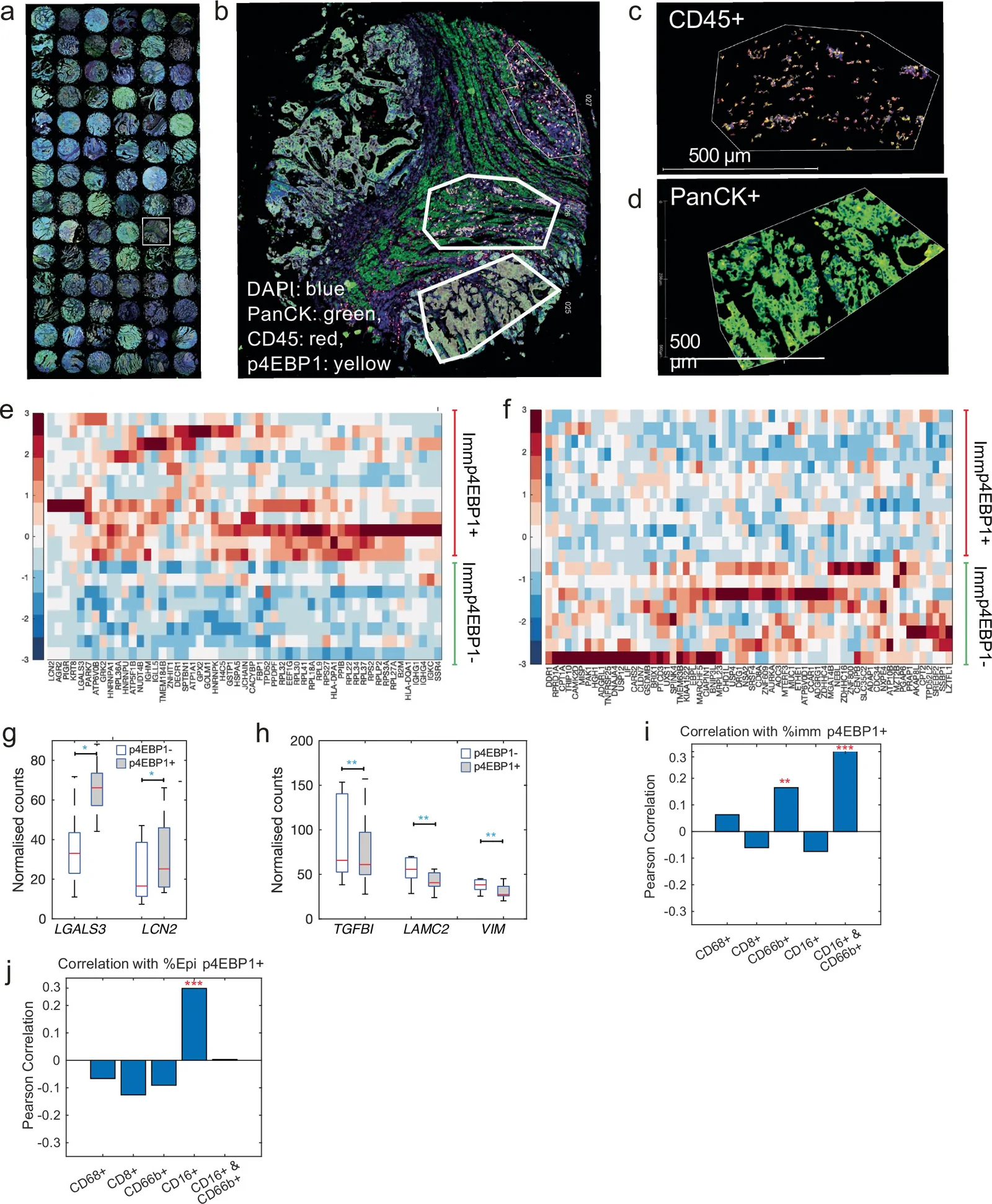
Validation using spatial transcriptomics and multiplexed imaging. **a** GeoMx Spatial profiling was performed on tumour microarray (TMA) stained for DAPI (blue), pan-cytokeratin (PanCK ) (green), p4EBP1 (yellow), and CD45 (red). **b** Inset of the region highlighted in (**a**). **c**, **d** Polygons are manually drawn to select immune cells (CD45+ ) versus epithelial regions (PCK+ ), followed by a thresholding approach in Digital Spatial Profile for detecting the relevant cells for spatial profiling. **e** Upregulated genes in immune regions in Imm^p4EBP1+^ tumours. **f** Downregulated genes in immune regions in Imm^p4EBP1+^ tumours. The colour scale ranges from blue (low gene expression values) to red (high gene expression values). **g** Normalised counts of genes associated with neutrophil activation in immune compartments (the star indicates KS *p-*value < 0.01). **h** Normalised counts of genes associated with epithelial to mesenchymal transition gene set in the epithelial compartment (two stars indicate KS *p-*value < 0.001). Box plots show the median (red line), interquartile range (box), whiskers extending to data points within 1.5 times the interquartile range, and outliers plotted individually (red plus markers), where grey boxes indicate Imm^p4EBP1+^ tumours. **i** Pearson correlation between the proportion of various immune cell types and the proportion of Imm^p4EBP1+^ cells based on 7-plex imaging data (*n* = 125 patients). **j** Pearson correlation between the proportion of various immune cell types and the proportion of Epi^p4EBP1+^ cells based on 7-plex imaging data. Two stars indicate a correlation *p*-value < 0.001, three stars indicate a correlation *p*-value < 0.0001 based on Pearson correlation.

To further validate the functional relevance of p4EBP1 activation in immune cells, we assessed spatial RNA expression of 18,677 genes in immune cells and epithelial cancer cells separately in an independent colorectal cancer patient cohort (Fig. 5a–d and ‘Methods’). Using the GeoMx Digital Spatial Profiler spatial transcriptomics platform, we harvested RNA from manually defined regions of interest (ROIs) in 20 tumours. We stained these tumours for p4EBP1 to assess p4EBP1 expression within immune cells. We identified various levels of p4EBP1 expression within tumours, which we classified as negative, low, medium, or high-expressing tumours. We confirmed that transcripts from CD45+ regions are well separated from transcripts from PCK+ regions (Supplementary Fig. 3a–c).

Analysis of gene expression in the immune compartment identified differential profiles between Imm^p4EBP1+^ and Imm^p4EBP1-^ tumours. Subsequent enrichment analysis using DAVID and GSEA revealed a significant enrichment of genes associated with immune activation in Imm^p4EBP1+^ tumours (Fig. 5e-f, ‘Methods’ and Supplementary Table 4-5). These include those involved in lymphoblast as well as immunoglobulin/major histocompatibility, such as *IGHM*, *IGLL5*, *HLA-DQA1*, and *HLA-DPA1*. Among the upregulated immune activation genes were the neutrophil activators *LCN2* (neutrophil gelatinase-associated lipocalin) and Galectin-3 (*LGALS3*) (Fig. 5g, *p*-value < 0.01). Moreover, immune cells in Imm^p4EBP1+^ tumours exhibited upregulation of genes involved in translation, including ribonucleoprotein (16 genes), RNA translation (13 genes), and RNA binding, which is expected given the role of 4EBP1 in translational regulation (Supplementary Tables 4 and 5; ‘Methods’). Interestingly, many of these translational genes are indicated to be significantly upregulated upon stimulation of neutrophils based on GSEA, including *RPL9, RPL27A, RPL18A, RPL34, RPS27, RPS2, RPS3A, RPLP2, RPL37, RPL22, RPL41*, and *EEF1G*. On the other hand, downregulation was observed in proliferation-associated genes, such as *AURKA* and *MISP,* as well as collagen receptors, such as *ADGRG1*, *ADGRL1*, *DDR1* and other adhesion proteins, such as *MUC1* (Fig. 5f). This suggests that p4EBP1 activity in immune cells is associated with activation of immune cells, particularly neutrophils, and suppression of proliferation and extracellular matrix interactions.

Next, we sought to identify if there were specific genes in the epithelial cancer cells that were associated with p4EBP1 activity in immune cells. Upregulated genes were enriched for several terms associated with immune response, including the gene ontology terms ‘regulation of immune system processes’ and ‘defence response’, and several terms associated with epithelial adhesions, such as Cadherin Binding (Supplementary Table 6). On the other hand, downregulated genes were enriched for several stemness-associated terms, including the ‘epithelial to mesenchymal transition’ (EMT) and ‘TNFA signalling via NFKB’ hallmark gene sets, as well as the gene ontology terms ‘cell motility’ and ‘cytoskeleton organisation’ (FDR < 0.05, and Supplementary Table 6). These include *TGFBI*, vimentin, and laminin subunit gamma-2 (*LAMC2*) (Fig. 5h, *p-*value < 0.001). Interestingly, there was little overlap between differentially expressed genes in epithelial and immune compartments (only 15% of genes). These results are consistent with our image-based proteomics data, suggesting that EMT-associated signalling is lower in Imm^p4EBP1+^ tumours.

### Validation of neutrophil enrichment in Imm^p4EBP1+^ tumours

To validate the association between neutrophils and p4EBP1 expression in immune cells, we stained for various immune markers using the Vectra Polaris platform (Supplementary Fig. 3d and ‘Methods’). This allowed quantifying the proportion of T-cells (CD8+ ), macrophages (CD68+ ), natural killer cells (CD16+) and neutrophils (CD16+ and CD66b+ ) in a TMA of 125 patients (Supplementary Fig. 3d). Interestingly, we observed a significant correlation between the proportion of p4EBP1+ immune cells and CD66b+ cells (*p*-value < 0.001) and even more significant correlation with CD16+ CD66b+ neutrophils (*p*-value < 0.0001 and Fig. 5i). On the other hand, the proportion of p4EBP1+ epithelial cells correlated most strongly with CD16+ cells implying differential roles for p4EBP1 in epithelial versus immune cells (Fig. 5j). These findings suggest an important link between p4EBP1 expression in immune cells and neutrophils, particularly the CD16+ CD66b+ sub-population.

### Integrative analysis links Imm^p4EBP1+^ signalling to colorectal cancer molecular subtypes

**Fig. 6:**
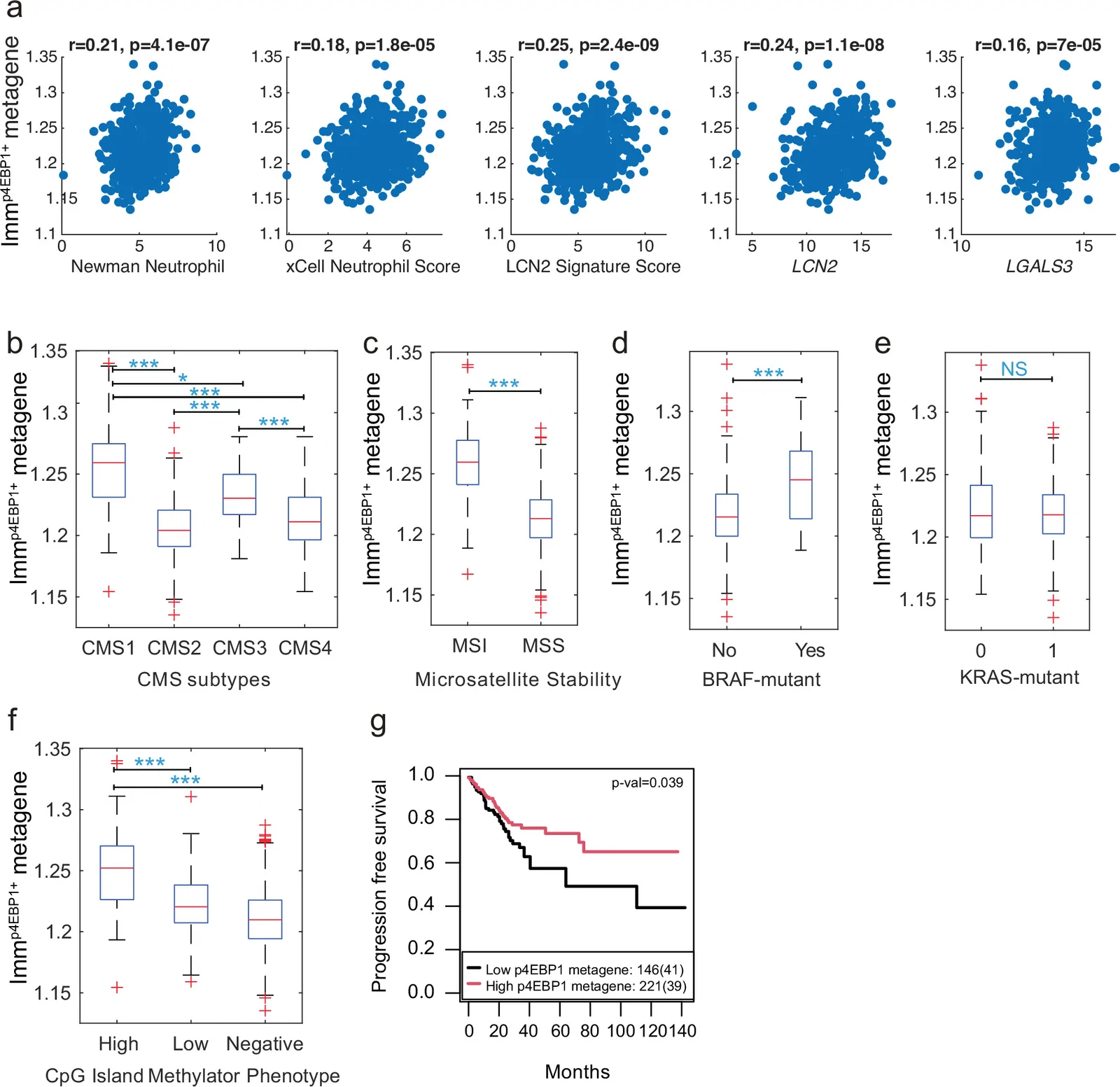
Definition of Imm^p4EBP1+^ metagene for integration with colorectal cancer TCGA data. **a** Pearson correlation between Imm^p4EBP1+^ metagene and neutrophil signatures, LCN2-associated neutrophil activation/degranulation signature, and expression levels of *LCN2* and *LGALS3*. b-f) Levels of Imm^p4EBP1+^ metagene based on: Colorectal Molecular Subtypes (CMS) (**b**), microsatellite instability (MSI) versus microsatellite stable (MSS) tumours (**c**), BRAF mutation status (**d**), KRAS mutation status (**e**), and methylation score based on CpG Island Methylator Phenotype (CIMP) (**f**). Box plots show the median (red line), interquartile range (box), whiskers extending to data points within 1.5 times the interquartile range, and outliers plotted individually (red plus markers). **g** Kaplan–Meier survival analysis shows that Imm^p4EBP1+^ metagene (red line) is associated with significantly higher progression-free survival in CMS 1-3 when compared to Imm^p4EBP1-^ metagene (black line) (*p*-value = 0.039). One star indicates *p*-value < 0.05, two stars indicate *p*-value < 0.001, and three stars indicate *p*-value < 0.0001. All pairwise comparisons were performed using the Kolmogorov–Smirnov test, except for comparisons across CMS1-4, where the Kruskal–Wallis test was used, followed by Dunn’s multiple-comparisons test with Sidak correction. NS not significant.

To enable further interrogation of Imm^p4EBP1+^ tumours, we sought to define a transcription-based metagene of Imm^p4EBP1+^ tumours. To ensure that this signature can be integrated with bulk RNA sequencing data from TCGA, we considered upregulated and downregulated genes in both immune and epithelial compartments based on the spatial transcriptomic analysis (‘Methods’). To increase confidence that the Imm^p4EBP1+^ metagene captured biology related to mTOR/4EBP1 signalling, we assessed its correlation with genes in the downstream mTOR-regulated translation-initiation/eIF4F axis, including *EIF4G1*, *EIF4A1* and *EIF4E1* (‘Methods’). The correlation was statistically significant but modest, supporting an association with the mTOR/4EBP1-regulated translation-initiation axis, while remaining consistent with the fact that TCGA bulk RNA-seq cannot directly capture phosphorylation-dependent or cell-type-specific p4EBP1 activity (Pearson correlation = 0.28, *p*-value = 5.3822e-12, and ‘Methods’). Consistent with our GeoMx findings, the Imm^p4EBP1+^ metagene positively correlated with several published neutrophil-based signatures (Fig. 6a, Pearson correlation >0.16, *p*-val < 0.0001). These include neutrophil identity signatures and an LCN2/granulation-associated signature, as well as expression values of *LCN2* and *LGALS3* (‘Methods’).

Interestingly, we found that the level of Imm^p4EBP1+^ metagene is significantly higher in CMS1 tumours, which are characterised by high immune activation, and in CMS3 tumours, compared to other CMS subtypes^21^ (Fig. 6b, Dunn’s test with Sidak correction adjusted *p*-value < 0.0001 and Supplementary Table 7). Moreover, Immp4EBP1+ metagene was significantly higher in all the signatures associated with CMS1, including microsatellite instability (MSI), high methylation rate as defined by CpG island methylator phenotype (CIMP), and BRAF mutations (Fig. 6c–f, *p*-value < 0.0001). On the other hand, Imm^p4EBP1+^ metagene has the lowest levels in CMS2 and CMS4 subtypes that are associated with WNT and TGF-β signalling, respectively. Interestingly, when excluding CMS4 patients, Imm^p4EBP1+^ metagene was predictive of better progression-free survival (*p*-value = 0.039 and Fig. 6g). These results agree with our findings based on multiplexed imaging and spatial transcriptomics and further support that TGF-β signalling could diminish the association between the Imm^p4EBP1+^ signature and patient prognosis.

## Discussion

The tumour microenvironment engages in numerous interactions with the tumour, influencing its progression and evolution. Typically, signalling in the tumour and the surrounding environment is often studied separately in the different compartments. Single-cell and spatial biology techniques provide enabling technologies for studying complex signalling in different cell types and compartments in the TME. In particular, multiplexed imaging offers the unique advantage of measuring spatial protein activities, including phosphorylation events, at the single-cell level. Here, we show how highly multiplexed imaging can advance our understanding of cell-type-specific mTOR cell signalling.

A key strength of our approach is that it captures p4EBP1-associated signalling within intact patient tumour architecture, preserving the spatial relationships between immune cells, epithelial cancer cells and the broader tumour microenvironment. This is important because the compartment-specific and shared epithelial-immune patterns identified here would be difficult to fully recapitulate in conventional in vitro systems, where stromal organisation, immune contexture and spatial signalling gradients are often lost.

In the multiplexed dataset, Imm^p4EBP1+^ tumours showed lower levels of the cell-cycle-associated protein CyclinB1, while spatial transcriptomics showed downregulation of proliferation-associated genes, including *AURKA* and *MISP*, together with reduced expression of genes linked to extracellular matrix interaction and adhesion. These observations are consistent with reduced proliferation-associated features in Imm^p4EBP1+^ tumours, but do not necessarily indicate global suppression of proliferation. They may instead reflect a distinct tumour state, potentially associated with an earlier stage of tumour progression compared with Imm^p4EBP1-^ tumours, consistent with the lower propotion of Imm^p4EBP1+^ tumours observed in Stage III tumours (Supplementary Fig. 1g). This pattern coexisted with higher expression of several ERK/MAPK-related proteins in both epithelial and immune cells, particularly in GNN-Cls 2. While ERK/MAPK activity can promote proliferation, its output is highly context dependent. ERK signalling has also been shown to enhance cellular heterogeneity, and MAPK activity can contribute to pathway crosstalk, DNA-damage responses, differentiation, immune activation and adaptive signalling downstream of microenvironmental stress^22–25^. Consistent with this, Imm^p4EBP1+^ tumours also showed higher protein levels of p53, MSH2 and MLH1. Future studies using murine colorectal cancer models could enable mechanistic perturbation of these signalling components using inhibitors, conditional knockout or CRISPR-based approaches. Such models could help determine whether the observed Imm^p4EBP1+^ phenotype reflects a causal tumour-microenvironment programme and explain its association with various biological functions and signalling activities.

Although Imm^p4EBP1+^ tumours exhibited higher MLH1 and MSH2 protein expression in our multiplex cohort, TCGA analysis demonstrated increased Imm^p4EBP1+^ metagene expression in MSI tumours. These findings are not necessarily contradictory. The positive association between Imm^p4EBP1+^ and mismatch-repair protein abundance may reflect shared upstream cellular programmes associated with context-dependent signalling, whereas the increased Imm^p4EBP1+^ metagene observed in MSI tumours may instead reflect the immune-rich tumour microenvironment characteristic of the MSI phenotype. Thus, the protein and transcriptomic observations may represent distinct biological mechanisms. Future studies integrating phosphoproteomics, transcriptomics, multiplex imaging and molecular MSI classification within the same patient cohort will be important to better understand these links.

Our findings indicate that a more favourable patient prognosis in Imm^p4EBP1+^ tumours is associated with a higher number of immune cells. Spatial transcriptomics analysis further supports that p4EBP1 expression in immune cells, including neutrophils, is linked to immune activation. Indeed, we found that p4EBP1 expression correlates with a higher number of neutrophils based on CD16 and CD66b markers. The function of neutrophils in the TME is highly complex, exhibiting anti-tumour as well as pro-tumour roles^26,27^. mTOR is known to play an important role in neutrophil function, including phagocytosis, metabolism, longevity, and chemotaxis^28–30^. These findings suggest that p4EBP1 is associated with neutrophil recruitment. Given the complexity of immune cell interactions in the immune microenvironment, more experiments will be needed to investigate the associated mechanisms.

GNN analysis allowed further refinement of anti-tumour Imm^p4EBP1+^ phenotype. Specifically, by integrating the influence of tissue morphology, cellular communication, and signalling states in different tissue compartments, GNN identified two distinct clusters of Imm^p4EBP1+^ tumours: GNN-Cls 2 and GNN-Cls 3. This classification led to improved prediction of patient outcomes, with only GNN-Cls 3 being associated with better overall survival. Spatial transcriptomics confirmed the downregulation of EMT and motility genes in epithelial cancer cells in Imm^p4EBP1+^ tumours, particularly genes in the TGF-β pathway such as vimentin and *TGFBI*. It is well-known that TGF-β induces vascularisation and extracellular matrix deposition, which consequently leads to suppressing immune function^31,32^. Indeed, we observe a reduction of vascularisation and fibronectin expression in Imm^p4EBP1+^ tumours (Fig. 3b–d). These results suggest that stemness and interlinked EMT programmes could suppress immune function by affecting several factors associated with decreased phosphorylation of 4EBP1 in immune cells.

To further investigate clinical relevance, we generated an Imm^p4EBP1+^ metagene and examined its association with CMS subtypes based on the TCGA dataset. This analysis further corroborated several observations derived from spatial transcriptomics and multiplexed immunofluorescence imaging. First, elevated Imm^p4EBP1+^ metagene levels in the immune-activated CMS1 confirm the link between p4EBP1 and immune response and activation in tumours. Notably, Imm^p4EBP1+^ metagene expression was also enriched in CMS3 tumours, indicating that its association is not restricted to the MSI-enriched CMS1 subtype and may reflect broader tumour and microenvironmental programmes. Additionally, the reduced Imm^p4EBP1+^ metagene levels in CMS4 are consistent with our finding of suppression of TGF-β signalling in Imm^p4EBP1+^ tumours. The prognostic advantage of Imm^p4EBP1+^ metagene in non-CMS4 tumours may explain the patient stratification in GNN-Cls 3 versus GNN-Cls 2. While MSI remains an established biomarker for immune checkpoint blockade, the enrichment of the Imm^p4EBP1+^ metagene in both CMS1 and CMS3 suggests that it may provide complementary biological information beyond MSI alone. Future studies with matched spatial protein profiling and transcriptomic data will be needed to validate the association between Imm^p4EBP1+^ and CMS subtypes and assess its potential as a biomarker for treatment, including immunotherapy.

While our study focused on p4EBP1 expression in immune cells, our results imply that p4EBP1 activity in epithelial cells could also have a positive effect on patient prognosis, depending on 4EBP1 expression. Such a positive effect could be attributed to the inhibition of 4EBP1 through its phosphorylation, as the expression of 4EBP1 in cancer cells counteracted the impact of p4EBP1 on survival. Moreover, a recent study has shown that bulk p4EBP1 expression in colorectal cancer is negatively correlated with fibroblast infiltration^12^. These findings, along with the role of 4EBP1 in immune cells, could explain the conflicting evidence on the effects of p4EBP1 on patient survival.

In summary, we propose that p4EBP1 has a dual function, supporting tumour growth and proliferation, yet mediating anti-tumour activity in immune cells when TGF-β signalling is low. Although many studies commented on these different aspects in diseased and healthy cells, to our knowledge, our study is the first to provide an integrated analysis of p4EBP1 in the immune microenvironment. As such it offers a potential explanation of the limited progress of mTOR inhibitors in the clinic, as many of those target p4EBP1, and therefore could have significant implications for the use of mTOR inhibitors in therapy. Furthermore, while preliminary, our results raise the possibility that p4EBP1 expression could serve as a prognostic biomarker. Most importantly, our study provides a framework for studying complex cell signalling while accounting for cell interactions within different TME components using multiplexed imaging and advanced analysis methods.

## Methods

### Multiplexed Dataset of 61 markers

We used a previously published multiplexed immunofluorescence dataset of colorectal cancer TMAs^1^. The dataset comprises 746 patients across three TMAs using 0.6 mm^2^ cores (up to 250 cores per TMA), including 383 males, 361 females and 2 patients with undefined sex. Survival data were available for 742 patients. Staining was performed using Cell DIVE™ technology as previously described (Leica Microsystems). After de-paraffinization and two-step antigen retrieval, the FFPE slides were stained with DAPI and imaged across all channels of interest to acquire background autofluorescence (AF) from the tissue. This was followed by indirect detection and/or direct conjugate antibody staining of up to 3 markers per round plus DAPI, dye deactivation, and repeat staining to collect images of all planned protein markers (Supplementary Table 1). Multiplexed images were automatically registered and processed for illumination correction and autofluorescence subtraction during each round of imaging using the Cell DIVE image processing workflow as previously reported in Gerdes et al., and Uttam et al.^1,3^.

### Image pre-processing and analysis

Our analysis builds on custom image processing and segmentation algorithms from Gerdes et al.^1^. In the present study, we identified different types of immune cells and marker expression was determined based on an adaptive thresholding method, with results qualitatively validated. Intensity thresholds were adjusted when a high level of noise was detected. A similar approach was used to identify cells positive for 4EBP1, p4EBP1, S6, pS6, and AKT. Stromal and epithelial regions were defined based on pan-cytokeratin (PCK), E-cadherin and SMA expression, utilising a combination of adaptive thresholding and dilation/erosion image operations. Each cell was then assigned either to the stromal or the epithelial region.

### Fibronectin and vessel analysis

Adaptive thresholding was applied to CD31 images to delineate vessel regions, which were subsequently analysed to obtain the vessel area. This was normalised to the stromal area within the tumour. We measured the pixel correlation based on the Grey-Level Co-occurrence Matrix as a proxy for fibronectin structure.

### Immune mTOR status

A tumour was defined to be Imm^p4EBP1+^ if at least 5% of immune cells in the tumour expressed p4EBP1. Based on this cut-off, ~70% of tumours were considered positive for Imm^p4EBP1+^ (Fig. 2d). Such a threshold ensures robustness to staining artifacts. We confirmed that the association between Imm^p4EBP1+^ and survival is not sensitive to a certain threshold, by testing several thresholds (Supplementary Fig. 1c, d). We found that Imm^p4EBP1+^ is consistently significantly associated with survival probability based on Kaplan–Meier analysis.

### Data normalisation

In addition to autofluorescence subtraction across different rounds, we normalised intensity features by calculating their z-score per slide to eliminate batch effect.

### Differential protein expression analysis

To identify signalling differences between Imm^p4EBP1+^ and Imm^p4EBP1-^ tumours, we performed Kolmogorov–Smirnov (KS) non-parametric test. The 30 most significantly different features in the immune compartment or epithelial compartment were considered. After removing p4EBP1, 29 markers remained, and the difference in average marker intensity was visualised in Fig. 3a.

Markers that are not typically expressed in immune cells were masked in Fig. 3a. Those were determined based on one or more of the following: 1) Qualitative assessment of protein expression in immune cell in colorectal cancer samples in Human Protein Atlas^33^, 2) Expression of the corresponding gene in immune cells based on immune proteome atlas^33^, or 3) literature documenting a role for the protein in immune function.

### Graph Neural Network (GNN) training

We employed GNNs, which are well-suited for graph-structured data, enabling it to account for spatial cell interactions using an adapted MesoGraph architecture^20^. The input to the GNN is a network of cells in a tumour along with their features. These networks were defined by representing each tumour as a graph where each cell is a node. Cell nodes were connected by edges if they were within 50 pixels of each other. Node features were defined based on signalling in various cell compartments, morphology, and neighbourhood features, excluding p4EBP1 measurements (273 features). The network is trained to predict two classes: 1) Imm^p4EBP1+^ tumours and 2) Immp4EBP1- tumours based on single-cell features and their spatial localisation. The network produces the probability of a tumour belonging to each class. Our approach also allows us to obtain the weights assigned by the network to each cell based on the predicted node class (high-scoring nodes). The network was trained using 5-fold cross-validation (*n *= 746).

### GNN-clustering

GNNExplainer was run for 200 epochs and four hops to rank the most predictive node features for each tumour. Features that have a score greater than 0.4 in at least 200 tumours were considered high-ranking features, resulting in 81 features. Tumours were clustered using hierarchical clustering with Euclidean distance and Ward linkage based on the z-scored ranks of node features. This clustering revealed three key clusters: GNN-Cls 1-3.

### Spatial Transcriptomics

Spatial transcriptomics was run on a tissue microarray of 125 cores and a core size of 1.5 mm. The cells were stained for DAPI, pan-cytokeratin, CD45 and p4EBP1. Due to the limitation of the GeoMx platform, only the most suitable cores were selected manually to harvest RNA from cancer versus the immune compartment based on CD45 and PCK expression. 20 cores were identified as having sufficient numbers of immune and epithelial cells (at least 50 cells per region of interest (ROI), per Digital Spatial Profiling guidelines). These were considered for further analysis.

### Digital spatial profiling (DSP)

Spatial transcriptomic profiling of the whole transcriptome was conducted on the NanoString GeoMx® platform according to the manufacturer’s guidelines and recommendations. Tissue was manually processed with 20 min heat-induced epitope retrieval at pH 9, followed by 15 min of proteinase K (1 μg/ml; Life Technologies) digestion at 37 °C. RNA was hybridised in situ with the GeoMx® Human Whole Transcriptome Atlas Probe Mix. For visualisation, tissues were stained using immunofluorescent primary antibodies against PanCK (clone: AE1 + AE3, conjugate: AF532; NanoString) and CD45 (clone: PD7/26 + 2B11, conjugate: AF594; NanoString), as well as unconjugated anti-p4EBP1 Thr37/46 (clone: 236B4; CellSignalling) and anti-rabbit secondary antibody (clone: ab150083; conjugate: AF647; Abcam). Nuclear staining was performed using SYTO13 (NanoString). Library preparation from the collected oligonucleotides was carried out according to the manufacturer’s instructions and sequenced on paired-end 150 NovaSeq 6000 with a sequencing depth of 1200 million reads. The Illumina fastq files were processed using the GeoMx NGS Pipeline to produce ‘.DCC’ files, uploaded to the GeoMx DSP analysis suite for filtering and normalisation. ROIs were excluded if they had <1000 raw reads, <80 aligned/stitched/trimmed reads or less than 50% sequencing saturation. For each transcript, outlying probes were excluded if their count geomean is <10% of the geomean of all probes for that transcript or if the probe is scored as an outlier in the Grubbs outlier test in >20% of ROIs. Transcripts or ROIs with expression over the threshold of the NanoString defined Limit of Quantification (LOQ) or user-defined threshold of 2 in less than 5% of transcripts or ROIs were filtered out. Transcript data was then Q3 normalised and analysed in MatLab (Supplementary Data 1).

### Spatial transcriptomics analysis

We performed differential expression analysis using rnaseqdiff^34^ in MatLab. We identified genes that have the highest log fold change with no or a small number of p4EBP1+ cells in the immune compartment versus medium to high numbers of p4EBP1+ cells in the immune compartment. Enrichment analysis was performed using DAVID and GSEA functional enrichment tools.

### 7-plex multiplexed panel for validating p4EBP1 role in neutrophil recruitment

Multiplex immunofluorescence staining was carried out on 5 µm formalin-fixed paraffin-embedded (FFPE) sections using the OPAL™ protocol (AKOYA Biosciences) on the Leica BOND RXm autostainer (Leica, Microsystems). Six consecutive staining cycles were performed using the following primary Antibody-Opal fluorophore pairings: 1) p-4EBP1 (CST, Cat# 2855)–Opal 480, 2) CD8 (Dako, Cat #M7103)–Opal 520, 3) CD66b (Biotechne, Cat#NB100-77808)–Opal 570, 4) CD68 (Dako, Cat# M0876)–Opal 620, 5) CD16 (Abcam, Cat# ab198507) Opal 690, 6) Pan-Cytokeratin (Dako, Cat# M3515)–Opal 780. Primary antibodies were incubated for 30 min and detected using the BOND™ Polymer Refine Detection System (DS9800, Leica Biosystems) as per manufacturer’s instructions, substituting the DAB for the Opal fluorophores, with a 10-min incubation time and without the haematoxylin step. Antigen retrieval at 100 °C for 20 min, as per standard Leica protocol, with Epitope Retrieval (ER) Solution 2 (AR9640, Leica Biosystems), was performed before each primary antibody was applied. Sections were then incubated for 10 min with spectral DAPI (FP1490, Akoya Biosciences) and the slides were mounted with VECTASHIELD® Vibrance™ Antifade Mounting Medium (H-1700-10, Vector Laboratories). Whole slide scans and multispectral images (MSI) were obtained on the AKOYA Biosciences Vectra® Polaris™. Batch analysis of the MSIs from each case was performed with the inForm 2.4.8 software provided. Finally, batched analysed MSIs were fused in HALO (Indica Labs), to produce a spectrally unmixed reconstructed whole tissue image, ready for analysis.

### Imm^p4EBP1+^ metagene

To define a metagene that reflects Imm^p4EBP1+^ signature, we considered differentially upregulated and downregulated genes in both immune and epithelial compartments based on the spatial transcriptomics analysis. The log ratio of median levels of upregulated genes to the median levels of downregulated genes was used to define the Imm^p4EBP1+^ metagene. To increase our confidence that this metagene reflects mTOR activity, we calculated its correlation with genes downstream of the translation-initiation/eIF4F axis: *EIF4G1*, *EIF4A1* and *EIF4E1*. We evaluated the association of Imm^p4EBP1+^ metagene with various clinical and phenotypic features in the TCGA colorectal cancer dataset per our previous work^35^. The threshold for Imm^p4EBP1+^ metagene status was defined based on its median expression in the TCGA dataset.

### Neutrophil signatures

Neutrophil-related transcriptional programmes were assessed using curated gene-expression signatures: 1) The neutrophil signature based on Newman et al.: *AIF1, APOBEC3A, AQP9, BTNL8, C5AR1, CAMP, CASP5, CCR3, CDA, CEACAM3, CFP, CHI3L1, CHST15, CLC, CREB5, CSF3R, CXCR1, CXCR2, DPEP2, EMR2, EMR3, FAM212B, FCGR3B, FFAR2, FPR1, FPR2, GPR97, HAL, HSPA6, IGSF6, IL18RAP, LILRA2, LILRB2, LST1, MAK, MEFV, MGAM, MMP25, MNDA, MXD1, NCF2, NFE2, P2RY13, P2RY14, PADI4, PGLYRP1, PLEKHG3, QPCT, REPS2, S100A12, STEAP4, TLR2, TLR8, TNFAIP6, TNFRSF10C, TREM1, TREML2, VNN1, VNN2*, and *VNN3*^36^. 2) xCell-based combining all neutrophil genes identified in Aran et al.: *CA4, CEACAM3, FCGR3B, CXCR1, CXCR2, PGLYRP1, MMP25, ZDHHC18, TRPM6, VNN3, P2RY13, CSF3R, FPR2, S100A12, TREM1, LILRB2, LILRA2, BTNL8, MEFV, BMX, AATK, GPR27*, and *IL18RAP*^37^. 3) The LCN2/Activation granulation-associated signature included *LCN2, MMP8, MMP9, S100A8, S100A9, S100A12, CAMP, TREM1, CXCL8* and *IL1B*^38^. These signatures were calculated as the median expression of genes within each signature and correlated with the Imm^p4EBP1+^ metagene using Pearson correlation. *LCN2* and *LGALS3* were also assessed individually because they were identified as neutrophil-associated/inflammatory genes in the GeoMx spatial transcriptomic analysis.

## Supplementary information

Supplementary information

Supplementary Data 1

Supplementary Data 1 Caption

## Data Availability

The spatial transcriptomic data generated in this study are provided in Supplementary Data 1. The multiplexed immunofluorescence dataset of 746 colorectal tumours analysed here was generated in a previous study and is available from that publication. Colorectal cancer TCGA data used for the metagene analysis are publicly available from the NCI Genomic Data Commons (https://portal.gdc.cancer.gov), and consensus molecular subtype assignments were obtained as described previously.

## Code availability

Code for the adapted GNN is available at https://github.com/sailem-group/GraphNets.
