# Supplementary material for: Decoding spatial mTOR signalling reveals distinct roles in the tumour microenvironment
Source: NPJ Precis Oncol. 2026 Sep 25;10:388. doi: 10.1038/s41698-026-01674-w (PMC13669363; doi:10.1038/s41698-026-01674-w)
Supplement: Supplementary file 1 [file 41698_2026_1674_MOESM1_ESM.pdf]

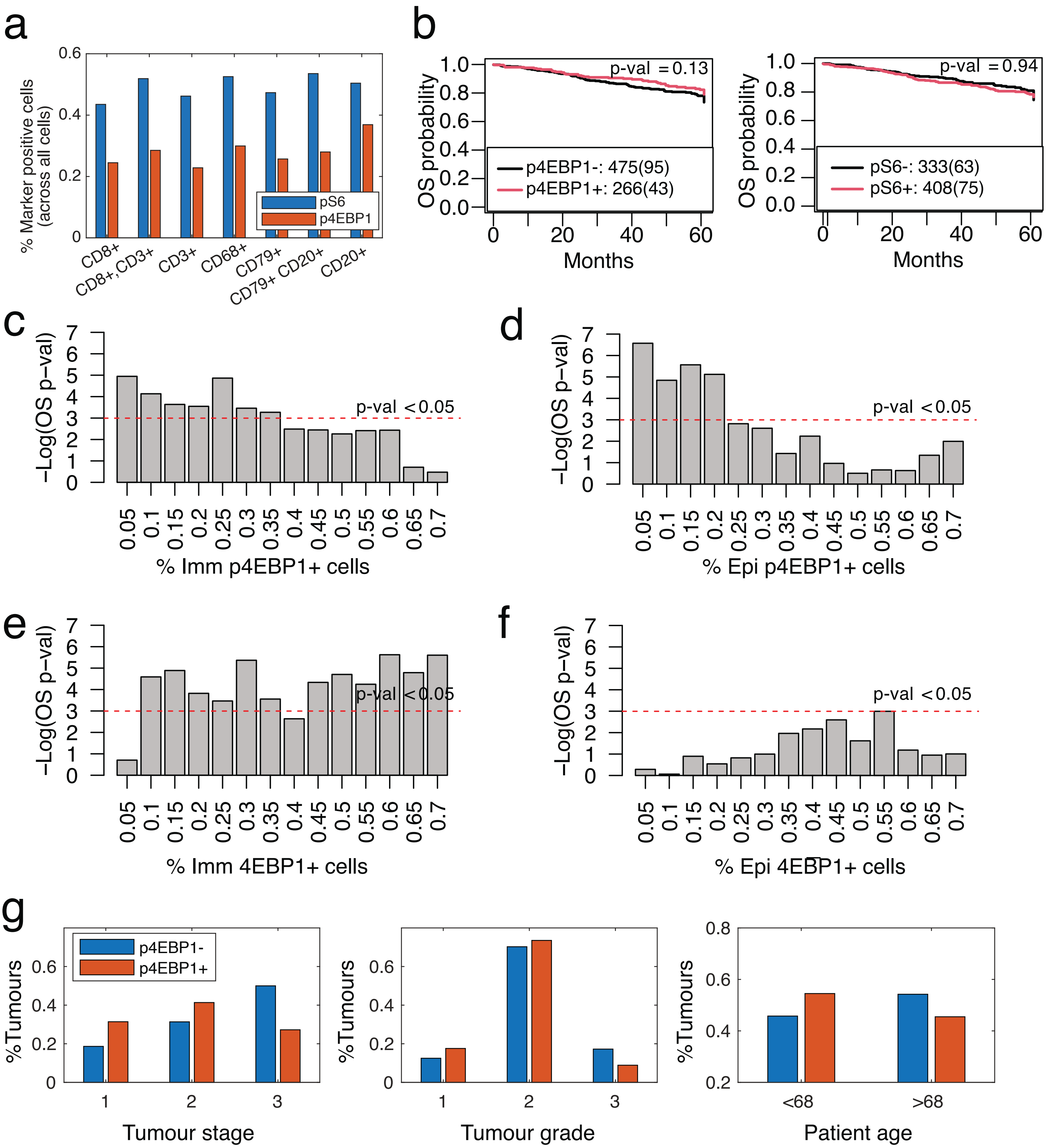

Supplementary Fig. 1. Association between mTOR and survival. a) Proportion of pS6+ (blue bars) and p4EBP1+ (red bars) cells across immune-cell types, calculated from pooled single cells across the cohort. b) Tumours with p4EBP1+ and pS6+ (red lines) signalling in stromal cells are not predictive of overall survival (OS) when compared to tumours with p4EBP1- and pS6- stroma (black lines). c-f) Shown is -log of *p-value* based on Kaplan Meier analysis of OS. The red line indicates *FDR* = 0.05 where higher values indicate higher statistical significance. c) p4EBP1 expression in immune cells is significantly associated with OS even when expressed in different percentages of immune cells confirming the robustness of this association. d) Same as (c) but in epithelial cells. e) When at least 5% of cells express p4EBP1, 4EBP1 expression in different percentages of immune cells generally does not impact the association between immune p4EBP1 and survival. f) The association between epithelial p4EBP1 and survival is dependent on the lack of 4EBP1 as 4EBP1 expression even in a small number of cells will diminish the correlation. g) Percentage of $Imm^{p4EBP1+}$ and $Imm^{p4EBP1-}$ tumours across different tumour stages, grades and age groups. The median age, defined by the 50th quantile, was used to categorise age into low and high groups, with ages over 68 classified as high. Red bars indicate $Imm^{p4EBP1+}$ and blue bars indicate $Imm^{p4EBP1-}$.

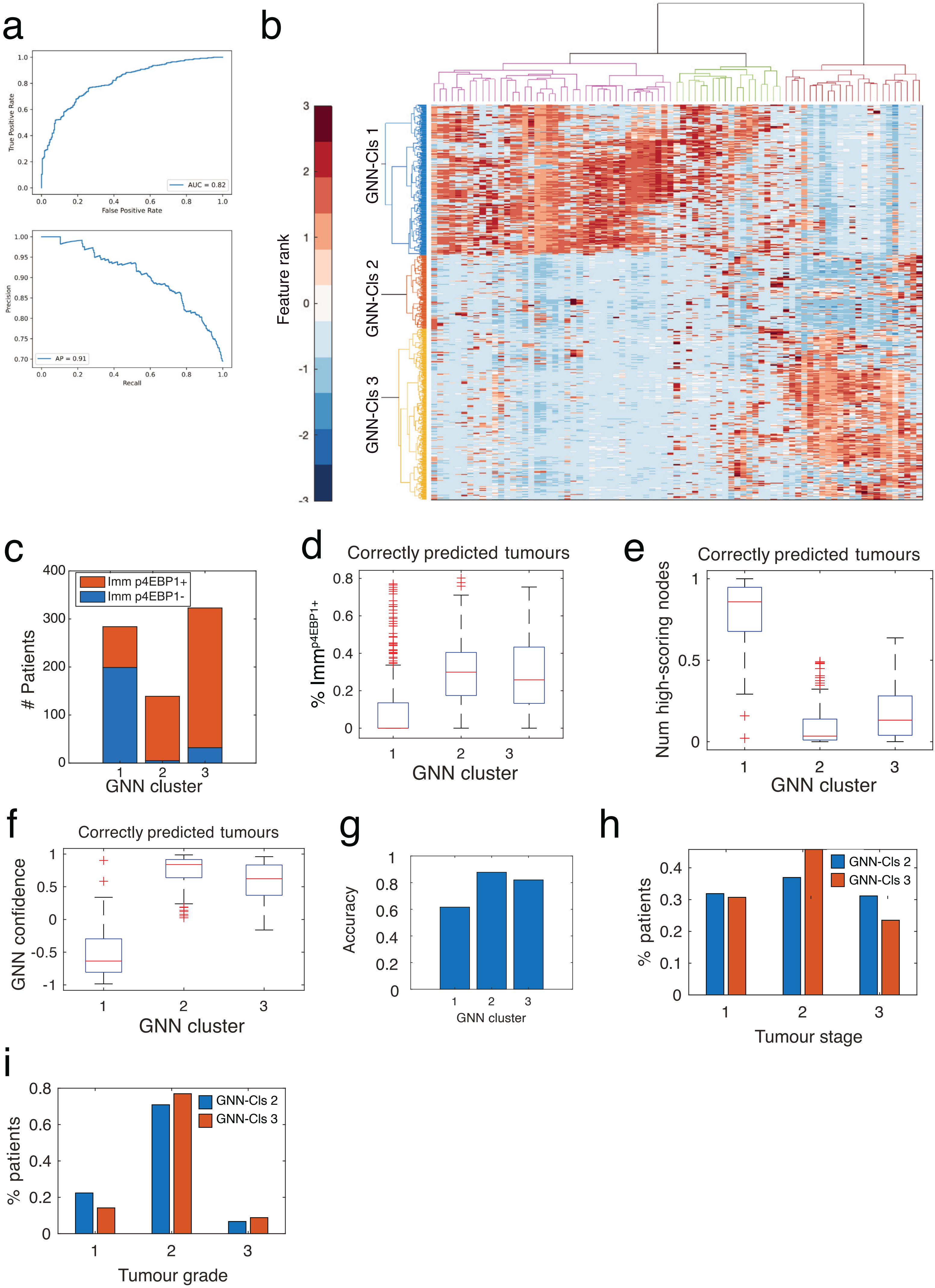
a
True Positive Rate
False Positive Rate
AUC = 0.82
Precision
Recall
AP = 0.91
b
Feature rank
GNN-Cls 1
GNN-Cls 2
GNN-Cls 3
c
Patients
Imm p4EBP1+
Imm p4EBP1-
GNN cluster
d
Correctly predicted tumours
% Imm$^{p4EBP1+}$
GNN cluster
e
Correctly predicted tumours
Num high-scoring nodes
GNN cluster
f
Correctly predicted tumours
GNN confidence
GNN cluster
g
Accuracy
GNN cluster
h
% patients
GNN-Cls 2
GNN-Cls 3
Tumour stage
i
% patients
GNN-Cls 2
GNN-Cls 3
Tumour grade

Supplementary Fig. 2. Analysis of GNN results. a) Area under the Curve (AUC) and Average Precision Curve for the GNN model. b) Clustering of features based on their GNN importance rank reveals that GNN predicts $Imm^{p4EBP1+}$ tumours based on a different set of features which distinguish three tumour groups named GNN-Cls. $Imm^{p4EBP1+}$ tumours can be divided into groups GNN-Cls 2 and 3. The colour scale ranges from blue (low feature importance) to red (high feature importance). c) Distribution of patients with $Imm^{p4EBP1+}$ tumours (red) compared to $Imm^{p4EBP1-}$ tumours (blue) across GNN-Cls groups d) GNN-Cls 2 and 3 have a comparable number of $Imm^{p4EBP1+}$ cells when considering correctly predicted tumours. e-g) GNN-Cls 2 and 3 have a comparable number of high-scoring cells (e), confidence prediction (f), and accuracy (g) in correctly predicted tumours. Box plots show the median (red line), interquartile range (box), whiskers extending to the most extreme data points within 1.5 times the interquartile range, and outliers plotted individually (red plus markers). h-i) Association between GNN-Cls 2 and 3 and tumour stage (h) and grade (i) is not significant (chi-square test *p-value*>=0.09). Blue bars indicate GNN-Cls 2 while red bars indicate GNN-Cls 3.

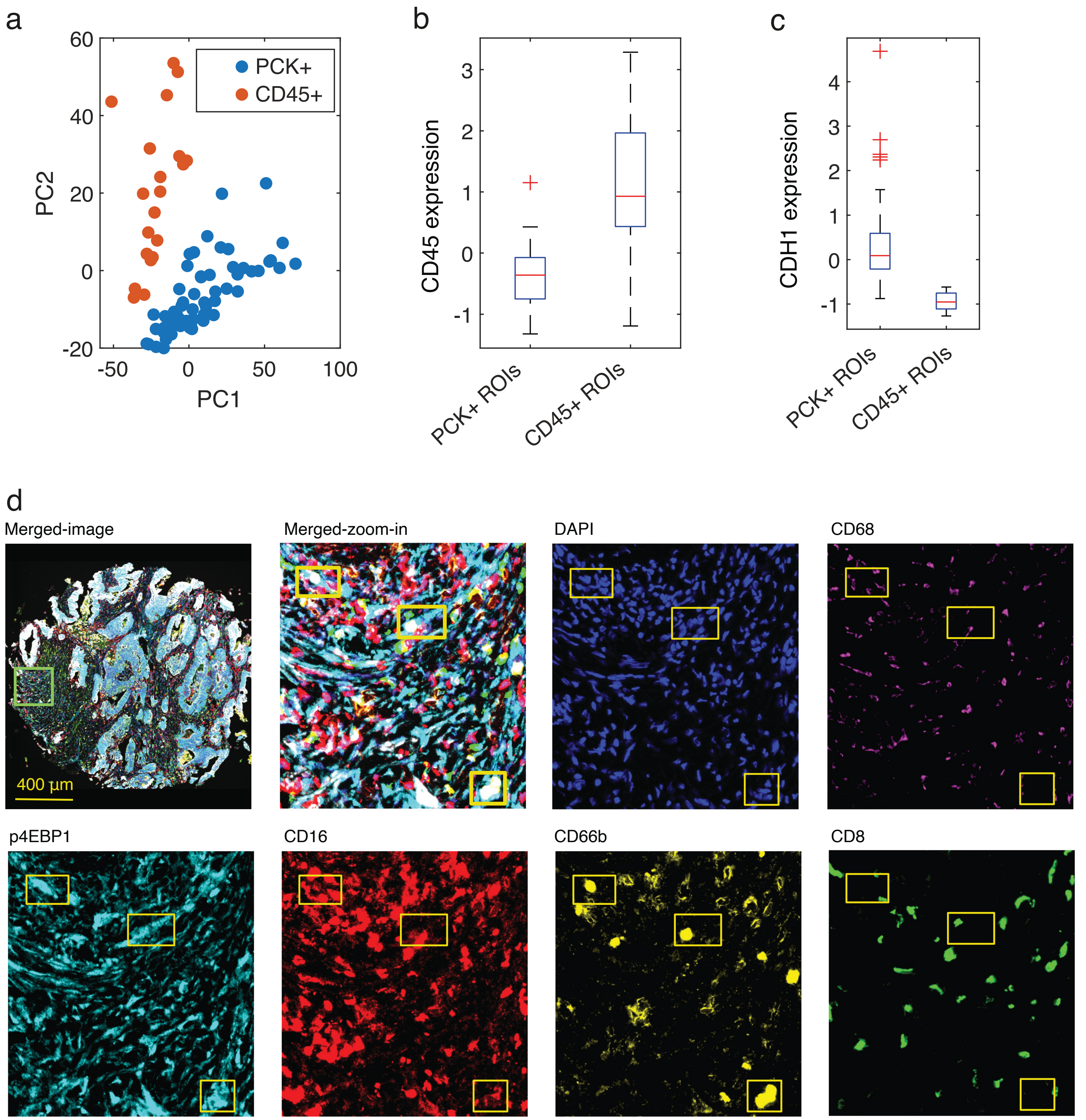

Supplementary Fig. 3. Validation using multiplexed and spatial data. a) RNA sequencing data of profiled regions are visualised in PCA space and shows that CD45+ regions (red filled circles) are separated from pan-cytokeratin+ (PCK+) regions (blue filled circles). b-c) Gene expression levels of *PTPRC* (CD45) and *CDH1* expression in CD45+ ROIs (Regions of interest) versus PCK+ ROIs respectively. Box plots show the median (red line), interquartile range (box), whiskers extending to the most extreme data points within 1.5 times the interquartile range, and outliers plotted individually (red plus markers). d) Example image of a tumour stained for DAPI (blue), PCK (white), p4EBP1 (cyan), CD8 (green), CD68 (magenta), CD16 (red), and CD66b (yellow). The green rectangle in the top left image shows the zoom-in region. Yellow rectangles in zoom-in images highlight CD16+CD66b+ cells expressing p4EBP1.

Supplementary Table 1: List of markers included in this study. More details are available in the original study.

Marker name	Protein	Gene name
4EBP1	4EBP1	EIF4EBP1
Akt	Akt	AKT1
ALDH1	ALDH1	ALDH1A1
beta_actin	beta_actin	ACTB
BetaCatenin	BetaCatenin	CTNNB1
CA9	CA9	CA9
CD163	CD163	CD163
CD20	CD20	MS4A1
CD3	CD3	CD3E
CD31	CD31	PECAM1
CD68	CD68	CD68
CD79	CD79	CD79A
CD8	CD8	CD8A
CK15	CK15	KRT15
CK19	CK19	KRT19
Claudin1	Claudin1	CLDN1
CleavedCaspase3	CleavedCaspase3	CASP3
cMET_epitomics	cMET_epitomics	MET
collagenIV	collagenIV	COL4A1, COL4A2, COL4A3, COL4A4, COL4A5, COL4A6
COX2	COX2	PTGS2
cyclinB1	cyclinB1	CCNB1
E_cad	E_cad	CDH1
EGFR	EGFR	EGFR
EPCAM	EPCAM	EPCAM
ERK	ERK	MAPK1, MAPK3
EZH2	EZH2	EZH2
Fibronectin	Fibronectin	FN1
FOXO1	FOXO1	FOXO1
FOXO3a	FOXO3a	FOXO3
Glut1	Glut1	SLC2A1
HER2	HER2	ERBB2
IHH	IHH	IHH
LaminA_C	LaminA_C	LMNA
MLH1	MLH1	MLH1
MSH2	MSH2	MSH2
NDRG1	NDRG1	NDRG1
p_p38MAPK	Phospho p38	MAPK14
p21	p21	CDKN1A
p4EBP1	Phospho 4EBP1	EIF4EBP1
p53	p53	TP53
pck26	pan cytokeratin	KRT1, KRT5, KRT6, KRT8
PCNA	PCNA	PCNA
pEGFR	Phospho EGFR	EGFR
pERK	Phospho ERK	MAPK1, MAPK3
pGSK3a	Phospho GSKa	GSK3A

pGSK3beta	Phospho GSKbeta	GSK3B
pMAPKAPK2	Phospho MAPKAPK2	MAPKAPK2
pMET_Y1349	Phospho MET_Y1349	HGFR
pNDRG1	Phospho NDRG1	NDRG1
pS6235	Phospho S6235	RPS6,
S6	S6	RPS6,
SMA	SMA	ACTA2
TKLP1	TKLP1	TKTL1
Vimentin	Vimentin	VIM
Wnt5	Wnt5	WNT5A
XCT	XCT	SLC7A11
Albumin	Albumin	Albumin
CD44v6	CD44v6	CD44
NaKATPase	NaKATPase	ATP1A1
PI3Kp110a	Phospoho PI3K	PIK3CA
PTEN	PTEN	PTEN

Supplementary Table 2: Cox Multivariate regression results.

Predictor	coef	exp(coef)	se(coef)	z	Pr(>|z|)
**Immp4EBP1+**	-0.464	0.629	0.174	-2.673	**0.007**
**Concordance**	0.552				
**Wald test - p-value**	**0.008**				

Predictor	coef	exp(coef)	se(coef)	z	Pr(>|z|)
**Immp4EBP1+**	-0.379	0.685	0.179	-2.114	**0.035**
**Grade**	0.485	1.624	0.171	2.833	**0.005**
**Concordance**	0.589				
**Wald test - p-value**	**0.001**				

Predictor	coef	exp(coef)	se(coef)	z	Pr(>|z|)
**Immp4EBP1+**	-0.139	0.871	0.178	-0.776	0.438
**Stage**	0.933	2.543	0.133	7.003	**2.50E-12**
**Concordance**	0.694				
**Wald test - p-value**	**1.21E-12**				

Predictor	coef	exp(coef)	se(coef)	z	Pr(>|z|)
**Immp4EBP1+**	-0.469	0.625	0.174	-2.701	**0.007**
**Lymph Nodes**	0.011	1.011	0.005	2.493	**0.013**
Concordance	0.640				
Wald test - p-value	**0.001**				

Predictor	coef	exp(coef)	se(coef)	z	Pr(>|z|)
**Immp4EBP1+**	-0.456	0.634	0.174	-2.624	**0.009**
**Age**	0.009	1.009	0.007	1.235	0.217
Concordance	0.577				
Wald test - p-value	**0.013**				

Predictor	coef	exp(coef)	se(coef)	z	Pr(>|z|)
**Immp4EBP1+**	-0.464	0.629	0.174	-2.670	**0.008**
**Gender**	0.028	0.979	0.170	-0.122	0.903
Concordance	0.549				
Wald test - p-value	**0.008**				

Predictor	coef	exp(coef)	se(coef)	z	Pr(>|z|)
**Immp4EBP1+**	-0.165	0.848	0.217	-0.761	0.447
**GNN-Cls**	-0.271	0.762	0.120	-2.262	**0.024**
Concordance	0.580				
Wald test - p-value	**0.002**				

Supplementary Table 3. List of top-ranked features by GNN explainers across GNN clusters.

Feature name	GNN-Cls1	GNN-Cls2	GNN-Cls3
'MedianMembpNDRG1'	1	0	0
'MedianNucpNDRG1'	1	0	0
'MedianCellpNDRG1'	1	0	0
'MedianCellpMET_Y1349'	1	0	0
'MedianCytpMET_Y1349'	1	0	0
'MedianMembpMET_Y1349'	1	0	0
'MedianCellp53'	1	0	0
'MedianCellcyclinB1'	1	0	0
'MedianCellCK19'	1	0	0
'MedianMembVimentin'	1	0	0
'MedianCytS6'	1	0	0
'MedianCytAlbumin'	1	0	0
'MedianCellCD8'	1	0	0
'MedianNucp_p38MAPK'	1	0	0
'MedianCellp_p38MAPK'	1	0	0
'MedianCellpGSK3a'	1	0	0
'MedianNucpGSK3a'	1	0	0
'MedianCellpMAPKAPK2'	1	0	0
'MedianMembpMAPKAPK2'	1	0	0
'MedianCytpMAPKAPK2'	1	0	0
'MedianNucpMAPKAPK2'	1	0	0
'MedianCellpS6235'	1	0	0
'MedianNucpS6235'	1	0	0
'MedianCellpERK'	1	0	0
'MedianNucpERK'	1	0	0
'MedianCytFibronectin'	1	0	0
'MedianCytpEGFR'	1	0	0
'MedianCytCOX2'	1	0	0
'MedianMembWnt5'	1	0	0
'MedianMembBetaCatenin'	1	0	0
'MedianCytE_cad'	1	0	0
'MedianCytNaKATPase'	1	0	0
'MedianCytCK19'	1	0	0
'MedianMembCD31'	1	0	0
'MedianCytVimentin'	1	0	0
'MedianCytBetaCatenin'	1	0	0
'MedianMembNaKATPase'	1	0	0
'MedianMembcMET_epitomics'	1	0	0
'MedianMembPI3Kp110a'	1	0	0
'MedianMembGlut1'	1	0	0
'MedianNucIHH'	1	0	0
'MedianCellIHH'	1	0	0
'MedianCytpERK'	1	0	0
'MedianMembCD79'	1	0	0
'MedianMembCK19'	1	0	0

'MedianCellBetaCatenin'	1	0	0
'MedianCell4EBP1'	1	0	0
'MedianNucDAPI'	1	0	0
'MedianCytALDH1'	1	0	0
'MedianMembERK'	1	0	0
'MedianCytEPCAM'	1	0	0
'MedianMembEPCAM'	1	0	0
'MedianMembpGSK3a'	1	0	0
'MedianCytp21'	1	0	0
'MedianCytpGSK3beta'	1	0	0
'MedianCellpGSK3beta'	1	0	0
'MedianNucFOXO3a'	1	0	0
'MedianCellFOXO3a'	1	0	0
'MedianMembFibronectin'	0	0	1
'NN_E2S_100'	0	0	1
'MedianMembLaminA_C'	0	0	1
'CD68'	0	0	1
'EpitheliumStroma'	0	0	1
MedianCytCD44v6'	0	0	1
'MedianMembIHH'	0	0	1
'MedianCytcMET_epitomics'	0	0	1
'AreaNuc2Cell'	0	0	1
'MedianMembE_cad'	0	0	1
'AreaCyt'	0	0	1
'MedianCytpck26'	0	0	1
'MedianMembAkt'	0	0	1
'MedianCytMLH1'	0	0	1
'MedianMembpEGFR'	0	0	1
'MedianMembXCT'	0	0	1
'MedianCytXCT'	0	1	1
'MedianCytNDRG1'	0	1	1
'MedianCytEGFR'	0	1	1
'MedianCytMSH2'	0	1	1
'MedianCytTKLP1'	0	1	1
'MedianCellFibronectin'	0	1	1
'MedianNucCD31'	0	1	1

Supplementary Table 4. Enrichment of genes upregulated in immune compartment based on DAVID

Translation

Category	Term	Count	%	PValue	Genes	List Total	Bonferroni	Benjamini	FDR
UP_KW_MOLECULAR_FUNCTION	KW-0687~Ribonucleoprotein	16	33.333	7.99E-16	RPL41, RPL32, RPL34, RPL22, RPL36A, HNRNPU, RPS3A, RPL9, HNRNPK, RPS27, RPL18A, RPL27A, RPLP2, RPS2, RPL37, HNRNPA1	34	2.33E-14	2.40E-14	2.40E-14
GOTERM_CC_DIRECT	GO:0022625~cytosolic large ribosomal subunit	10	20.833	8.27E-15	RPL41, RPL32, RPL18A, RPL34, RPL27A, RPL22, RPL36A, RPLP2, RPL37, RPL9	47	1.26E-12	6.32E-13	5.79E-13
GOTERM_MF_DIRECT	GO:0003735~structural constituent of ribosome	13	27.083	1.51E-14	RPL41, RPL32, RPL34, RPL22, RPL36A, RPS3A, RPL9, RPS27, RPL18A, RPL27A, RPLP2, RPS2, RPL37	46	2.54E-12	2.53E-12	2.52E-12
UP_TISSUE	Lymphoblast	15	31.25	6.91E-14	RPL32, RPL22, HNRNPU, RPS3A, RPL9, EEF1G, HNRNPK, RPS27, ATP5F1B, RPL18A, RPL27A, RPLP2, RPS2, DECR1, H4C5	46	6.01E-12	6.01E-12	5.25E-12
GOTERM_BP_DIRECT	GO:0006412~translation	13	27.083	1.14E-13	RPL41, RPL32, RPL34, RPL22, RPL36A, RPS3A, RPL9, RPS27, RPL18A, RPL27A, RPLP2, RPS2, RPL37	47	5.24E-11	2.62E-11	2.62E-11
KEGG_PATHWAY	hsa03010:Ribosome	13	27.083	5.02E-13	RPL41, RPL32, RPL34, RPL22, RPL36A, RPS3A, RPL9, RPS27, RPL18A, RPL27A, RPLP2, RPS2, RPL37	33	4.72E-11	4.72E-11	4.72E-11
KEGG_PATHWAY	hsa05171:Coronavirus disease - COVID-19	13	27.083	2.69E-11	RPL41, RPL32, RPL34, RPL22, RPL36A, RPS3A, RPL9, RPS27, RPL18A, RPL27A, RPLP2, RPS2, RPL37	33	2.53E-09	1.27E-09	1.27E-09
GOTERM_MF_DIRECT	GO:0003723~RNA binding	20	41.667	2.60E-10	NUDT4B, RPL41, RPL32, RPL34, RPL22, RPL36A, HNRNPU, RPS3A, RPL9, LGALS3, HNRNPK, RPS27, RPL18A, RPL27A, RPS2, RPL37, PPIB, HNRNPA1, SPTBN1, H4C5	46	4.36E-08	2.18E-08	2.17E-08

Cancer

Category	Term	Count	%	PValue	Genes	List Total	Bonferroni	Benjamini	FDR
UP_TISSUE	Cervix carcinoma	24	50	6.39E-04	TPD52, TMEM184B, HSPA5, RPL34, RPL22, KRT8, HNRNPU, RPS3A, CACYBP, PARK7, ATP1A1, LGALS3, HNRNPK, RPS27, GRK2, RPL18A, PPDPF, RPL27A, RPLP2, RPS2, RPL37, HNRNPA1, SPTBN1, H4C5	46	0.05412477	0.00927114	0.00809892
UP_TISSUE	Colon carcinoma	8	16.667	3.84E-04	HNRNPK, HSPA5, GSTP1, KRT8, HNRNPU, CACYBP, HNRNPA1, RPL9	46	0.03288005	0.00668527	0.00584

Immune-related

Category	Term	Count	%	PValue	Genes	List Total	Bonferroni	Benjamini	FDR
SMART	SM00407:IGc1	4	8.3333	2.20E-04	IGHM, IGLL5, HLA-DQA1, HLA-DPA1	18	0.00527155	0.00528491	0.00528491
UP_KW_BIOLOGICAL_PROCESS	KW-0391~Immunity	5	10.417	0.07295111	IGHM, LGALS3, LCN2, HLA-DQA1, HLA-DPA1	21	0.92943451	0.63832218	0.63832218
GOTERM_BP_DIRECT	GO:0045087~innate immune response	5	10.417	0.05512832	IGHM, LGALS3, IGLL5, LCN2, JCHAIN	47	1	1	1

Supplementary Table 5. Enrichment of genes upregulated in immune compartment based on GSEA

Gene Set Name	# Genes in Gene Set (K)	# Genes in Overlap (k)	k/K	p-value	FDR q-value	Genes
**GSE2405_0H_VS_24H_A_PHAGOCYTOPHILUM_STIM_NEUTROPHIL_UP**	200	15	0.075	1.80E-22	1.95E-18	RPL9, RPL27A, RPL18A, RPL34, RPS27, RPS2, RPS3A, RPLP2, RPL37, RPL22, RPL41, EEF1G
**GOBP_PEPTIDE_METABOLIC_PROCESS**	954	15	0.0157	2.01E-12	2.18E-09	RPL9, RPL27A, RPL18A, RPL34, RPS27, RPS2, RPS3A, RPLP2, RPL37, RPL22, RPL41, EEF1G, RPL32, RPL36A, HNRNPU
**GOCC_CELL_SUBSTRATE_JUNCTION**	435	9	0.0207	7.76E-09	5.44E-06	RPL9, RPS2, RPS3A, RPLP2, RPL22, HNRNPK, HSPA5, PPIB, HSPA1A
**GOCC_ANCHORING_JUNCTION**	899	11	0.0122	3.29E-08	1.74E-05	RPL9, RPS2, RPS3A, RPLP2, RPL22, HNRNPK, HSPA5, PPIB, HSPA1A, PARK7, KRT8
**GOBP_REGULATION_OF_RNA_SPLICING**	181	6	0.0331	1.85E-07	8.56E-05	HNRNPK, HSPA1A, HNRNPU, LGALS3, HNRNPA1, PTBP1, SUPT7L
**GOBP_RESPONSE_TO_CYTOKINE**	932	9	0.0097	4.59E-06	1.33E-03	HSPA1A, HSPA5, HNRNPU, KRT8, ATP5F1B, HLA-DPA1, GSTP1, CACYBP, CXCL5
**GOBP_REGULATION_OF_RESPONSE_TO_ENDOPLASMIC_RETICULUM_STRESS**	82	4	0.0488	5.04E-06	1.42E-03	HSPA1A, HSPA5, PARK7, AGR2
TEAD2_TARGET_GENES	1528	15	0.0098	1.41E-09	1.09E-06	PARK7, HSPA5, KRT8, ATP5F1B, CACYBP, SUPT7L, RPS3A, RPLP2, RPL22, RPL27A, RPL18A, EEF1G, RPL32, ZNHIT1, KRT20
**GOMF_ANTIOXIDANT_ACTIVITY**	83	4	0.0482	5.29E-06	1.48E-03	GSTP1, PARK7, FABP1, GPX2, HSPA1A, HSPA5

Supplementary Table 6. Enrichment of differentially expressed genes between Imm-p4EBP1+ and Imm-p4EBP1- tumours in epithelial compartment

Downregulated genes in epithelial compartment

Gene Set Name	# Genes in	# Genes in Overlap	k/K	p-value	FDR q-value	Genes
HALLMARK_APICAL_JUNCTION	200	5	0.025	1.08E-6	2.84E-3	TGFBI, LAMC2, LAMB3, COL17A1, SHC1
HALLMARK_EPITHELIAL_MESENCHYMAL_TRANSITION	200	5	0.025	1.08E-6	2.84E-3	TGFBI, LAMC2, VIM, PMEPA1, SPP1
HALLMARK_TNFA_SIGNALING_VIA_NFKB	200	5	0.025	1.08E-6	2.84E-3	LAMB3, PMEPA1, MARCKS, CCL20, TSC22D1
GOMF_EXTRACELLULAR_MATRIX_BINDING	55	3	0.0545	1.79E-5	1.34E-2	TGFBI, LAMC2, LAMB3, COL17A1
GOBP_CELL_MOTILITY	1813	9	0.005	2.92E-5	1.71E-2	CCL2, SMOC2, VIL, LAMC2, EPHB3, DNAJA4, HOXB9, CEMIP, CXCL5
GOBP_CELLULAR_RESPONSE_TO_STRESS	1966	9	0.0046	5.49E-5	2.42E-2	KRT20, CCDC13, SUPT7L
GOBP_CYTOSKELETON_ORGANIZATION	1527	8	0.0052	5.99E-5	2.52E-2	SHC1, VIM, MARCKS, VIL1, ATRX, MUC20, SLC6A6, CA9

Upregulated genes in epithelial compartment

Gene Set Name	# Genes in Gene Set (K)	# Genes in Overlap (k)	k/K	p-value	FDR q-value	Genes
GOCC_NUCLEOSOME	134	15	0.1119	2.26E-24	1.19E-20	H2BC11, H3C10, H3C2, H4C15, H4C12, H2BC9, H3C15, H3C13, H2AC21, H2AC19, H2AC20, H2AC12, H2AC11, H1-2, H1-4
GOMF_CADHERIN_BINDING	335	9	0.0269	2.19E-9	1.89E-6	H3C10, H3C2, OLFM4, HSPA8, PRDX1, EEF1G, HSPA5, LDHA, EEF1D
GOBP_CELL_KILLING	225	7	0.0311	5.5E-8	2.89E-5	PRDX1, H2BC11, IGHG1, HLA-DRA, APOL1, LYZ, PLA2G2A
GOBP_REGULATION_OF_IMMUNE_SYSTEM_PROCESS	1553	14	0.009	7.13E-8	3.41E-5	IGHG1, HLA-DRA, HSPA8, IGHG3, IGHG2, IGHG4, IGKC, HLA-DQA1, S100A14, EREG
GOBP_LYMPHOCYTE_MEDIATED_IMMUNITY	358	8	0.0223	7.49E-8	3.42E-5	PRDX1, HLA-DRA, IGHG1, IGHG3, IGHG2, IGHG4, IGKC, IGLL5
GOBP_DEFENSE_RESPONSE	1855	14	0.0075	6.13E-7	1.73E-4	IGHG1, HSPA8, IGHG3, IGHG2, IGHG4, S100A14, EREG, PRDX1, H2BC11, APOL1, LYZ, PLA2G2A, GSTP1, AGR2
HALLMARK_GLYCOLYSIS	200	6	0.03	6.41E-7	1.73E-4	HSPA5, LDHA, AKR1A1, GFPT1, PYGB, GFUS
HALLMARK_MTORC1_SIGNALING	200	6	0.03	6.41E-7	1.73E-4	HSPA5, LDHA, HSP90B1, SCD, RPN1, PRDX1
GOCC_NUCLEAR_OUTER_MEMBRANE_ENDOPLASMIC_RETICULUM_MEMBRANE_NETWORK	1212	9	0.0074	8.87E-5	1.24E-2	HLA-DRA, HLA-DQA1, HSP90B1, UBB, PLA2G2A, HSPA5, SCD, RPN1, TRAM1
GOCC_ENDOCYTIC_VESICLE	348	5	0.0144	1.93E-4	2.3E-2	EREG, HLA-DRA, HLA-DQA1, HSP90B1, UBB
GOBP_PEPTIDE_METABOLIC_PROCESS	997	8	0.008	1.33E-4	1.69E-2	EEF1G, EEF1D, RPS3A, RPS13, RPS8, RPL27A, EIF3F, YBX1

Supplementary Table 7. Dunn-Sidak adjusted pairwise comparisons of immune p4EBP1 metagene scores across CMS groups

Group 1	Group 2	Lower CI	Difference	Upper CI	Adjusted p-value
CMS1	CMS2	162.261	214.0524	265.8438	0
CMS1	CMS3	3.6544	67.6703	131.6862	0.0321
CMS1	CMS4	118.8284	174.0175	229.2067	6.66E-16
CMS2	CMS3	-199.232	-146.3821	-93.5322	1.90E-12
CMS2	CMS4	-81.7591	-40.0348	1.6894	0.0675
CMS3	CMS4	50.1636	106.3472	162.5308	3.81E-06

Supplementary Data 1. RNA count data from GeoMx profiling of epithelial and immune compartments across 20 colorectal cancer patients. Only genes that passed quality control are included.
