# Supplementary material for: Decoding spatial mTOR signalling reveals distinct roles in the tumour microenvironment
Source: NPJ Precis Oncol. 2026 Sep 25;10:388. doi: 10.1038/s41698-026-01674-w (PMC13669363; doi:10.1038/s41698-026-01674-w)
Supplement: Supplementary file 2 [file 41698_2026_1674_MOESM3_ESM.docx]

**Supplementary Data 1. RNA count data from GeoMx profiling of epithelial and immune compartments across 20 colorectal cancer patients. Only genes that passed quality control are included.**
